# ER, Mitochondria, and ISR Regulation by mt‐HSP70 and ATF5 upon Procollagen Misfolding in Osteoblasts

**DOI:** 10.1002/advs.202201273

**Published:** 2022-08-21

**Authors:** Laura Gorrell, Elena Makareeva, Shakib Omari, Satoru Otsuru, Sergey Leikin

**Affiliations:** ^1^ Eunice Kennedy Shriver National Institute of Child Health and Human Development (NICHD) National Institutes of Health (NIH) Bethesda MD 20892 USA; ^2^ Department of Biomedical Engineering Rensselaer Polytechnic Institute Troy NY 12180 USA; ^3^ NICHD NIH Bethesda MD 20892 USA; ^4^ Department of Orthopaedics University of Maryland School of Medicine Baltimore MD 21201 USA; ^5^ Sanford Burnham Prebys Medical Discovery Institute La Jolla CA 92037 USA

**Keywords:** ATF5, cell stress, collagen, osteoblast, osteogenesis imperfecta, HSPA9/mt‐Hsp70/GRP75

## Abstract

Cellular response to protein misfolding underlies multiple diseases. Collagens are the most abundant vertebrate proteins, yet little is known about cellular response to misfolding of their procollagen precursors. Osteoblasts (OBs)—the cells that make bone—produce so much procollagen that it accounts for up to 40% of mRNAs in the cell, which is why bone bears the brunt of mutations causing procollagen misfolding in osteogenesis imperfecta (OI). The present study of a G610C mouse model of OI by multiple transcriptomic techniques provides first solid clues to how OBs respond to misfolded procollagen accumulation in the endoplasmic reticulum (ER) and how this response affects OB function. Surprisingly, misfolded procollagen escapes the quality control in the ER lumen and indirectly triggers the integrated stress response (ISR) through other cell compartments. In G610C OBs, the ISR is regulated by mitochondrial HSP70 (mt‐HSP70) and ATF5 instead of their BIP and ATF4 paralogues, which normally activate and regulate ISR to secretory protein misfolding in the ER. The involvement of mt‐HSP70 and ATF5 together with other transcriptomic findings suggest that mitochondria might initiate the ISR upon disruption of ER‐mitochondria connections or might respond to the ISR activated by a yet unknown sensor.

## Graphical abstract

Multimodal transcriptomic analysis of osteoblasts in G610C mice reveals noncanonical cellular response to endoplasmic reticulum (ER) disruption by misfolded mutant procollagen, which is mediated by mt‐HSP70 (*Hspa9*) and ATF5 (*Atf5*) instead of their BIP (*Hspa5*) and ATF4 (*Atf4*) paralogues. Unlike canonical ER stress initiated by ER lumen chaperones and IRE1, ATF6, and PERK membrane receptors, this response appears to involve disruption of ER‐mitochondria contacts.

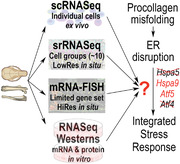

## 1 Introduction

Cellular malfunction caused by disruptions in protein homeostasis underlies numerous protein misfolding diseases (proteinopathies), which range from rare heritable developmental disorders in children to age‐related diseases.^[^ ^1^ ^]^ Collagenopathies caused by misfolding of collagen precursors (procollagens) in the endoplasmic reticulum (ER) are particularly puzzling and important.^[^ ^2^ ^]^ Collagens are by far the most abundant structural proteins in all vertebrates. Procollagen misfolding leads to heritable skeletal dysplasias and muscular dystrophy and likely contributes to fibrosis, arthritis, osteoporosis, and other common ailments.^[^ ^2^ ^]^ Like all secretory proteins, procollagens are folded in the ER before they are trafficked through Golgi and exported from the cell. ER disruption by their misfolding and its consequences for the cellular function are potential treatment targets for normalizing collagen homeostasis and reducing cellular malfunction, yet the molecular pathways involved in this disruption are poorly understood.

The best studied cellular response to ER disruption by misfolded secretory proteins (ER stress) is an unfolded protein response usually referred to as UPR.^[^ ^3^ ^]^ Hereafter, we refer to it as ER‐UPR to distinguish it from a distinct mitochondrial UPR (mt‐UPR).^[^ ^4^ ^]^ In ER‐UPR, misfolded protein accumulation in the ER lumen is detected by BIP/GRP78 and other luminal chaperones, activating stress receptors IRE1, ATF6, and/or PERK in the ER membrane and multiple cellular adaptation pathways downstream of these receptors. The cell then attempts to rescue ER homeostasis through ER associated proteasomal degradation (ERAD) of some misfolded proteins, delivery of other misfolded proteins to lysosomes (autophagy), upregulation of ER lumen chaperones, and suppression of secretory protein synthesis.

BIP encoded by the *HSPA5* gene is an HSP70 family chaperone in the ER lumen (ER‐HSP70) and a master regulator of ER‐UPR with multiple proposed functions.^[^ ^3^ ^]^ The two functions of BIP most relevant for the present study are: a) BIP promotes protein folding by binding to exposed hydrophobic surfaces on unfolded/misfolded chains. b) It prevents activation of IRE1, ATF6, and PERK by binding to them. Sequestration of BIP by accumulating misfolded proteins releases it from these receptors and activates them. Upregulation of BIP and recovery of ER homeostasis allows it to return to the ER‐UPR receptors and silence them.

Suppression of the secretory protein synthesis downstream of ER‐UPR is regulated by the integrated stress response (ISR).^[^ ^5^ ^]^ The ISR is characterized by EIF2*α* phosphorylation, which downregulates translation of most proteins yet upregulates translation of ATF4. The transcription factor ATF4 upregulates *HSPA5* downstream of the ER‐UPR and many ISR genes, including *EIF4EBP1*.^[^ ^6^ ^]^ EIF4E binding protein 1 encoded by *EIF4EBP1* inhibits formation of the EIF4F translation initiation complex, further reducing translation of secretory proteins and helping to restore ER homeostasis.^[^ ^7^ ^]^ ATF4 both regulates transcription and is transcriptionally regulated by CHOP, which is a cell survival/apoptosis factor encoded by *DDIT3*. In the ER‐UPR, EIF2*α* is phosphorylated by the PKR‐like ER kinase PERK. However, in other ISR branches, EIF2*α* may be phosphorylated by three other EIF2*α* kinases, PKR, GCN2, or HRI.^[^ ^5^ ^]^

Misfolding of secretory proteins may also disrupt the ER and cause cellular malfunction without ER‐UPR. For instance, misfolding and polymerization of *α*1‐antitrypsin Z variant (ATZ) in hepatocytes is not recognized by BIP and does not activate ER‐UPR, although it sensitizes the cells to ER‐UPR.^[^ ^8^ ^]^ Instead, “ER overload” response to ATZ activates NF*κ*B signaling, which also occurs upon misfolding of mutant CFTR protein causing cystic fibrosis and upon accumulation of some viral proteins in the ER.^[^ ^9^ ^]^ Nonetheless, ER‐UPR is activated by other *α*1‐antitrypsin mutations,^[^ ^10^ ^]^ ATZ in peripheral blood monocytes,^[^ ^11^ ^]^ and other viral proteins.^[^ ^12^ ^]^ ER‐UPR and ER overload appear to be distinct responses to ER disruption, which may operate separately or together depending on the protein, its misfolded conformation, and the cell type.^[^ ^8a^ ^]^

Triple helix misfolding in type I procollagen may trigger yet another type of cellular response, lacking the characteristic ER‐UPR and ER overload features.^[^ ^13^ ^]^ Type I is the most common collagen (collagen I), which forms the structural scaffold of bone, skin, and other tissues.^[^ ^14^ ^]^ Its procollagen precursor is a heterotrimer of two pro*α*1(I) and one pro*α*2(I) chains encoded by *COL1A1* and *COL1A2* genes. The chains fold together into a 300‐nm‐long triple helix bounded by N‐ and C‐terminal propeptides, which are cleaved upon secretion from cells and prior to assembly of mature collagen into extracellular matrix (ECM) fibers. Procollagen folding requires a Gly in every third position of the triple helix and presents a major challenge for cells.^[^ ^15^ ^]^ A substitution of just one out of 1014 obligatory Gly‐s in human collagen I causes osteogenesis imperfecta (OI) characterized by bone fragility and skeletal deformities.^[^ ^16^ ^]^ Gly substitutions in collagen I are responsible for over 80% of severe OI cases. Misfolded procollagen with Gly substitutions accumulates in and disrupts the ER of collagen‐producing cells. The resulting malfunction of osteoblasts (OBs)—cells that produce massive amounts of collagen I to make bone—is a major factor in bone pathology.^[^ ^13^, ^17^ ^]^ Understanding how OBs respond to the ER disruption by misfolded procollagen is important for identifying new therapeutic approaches to OI and potentially even common, age‐related osteoporosis.^[^ ^15^ ^]^

Triple helix misfolding in collagen I caused by Gly substitutions was found to occur without sequestering BIP (probably because this misfolding does not expose hydrophobic surfaces).^[^ ^18^ ^]^ Consistently, no BIP upregulation or other evidence of the ER‐UPR was found in vivo and in cultured OBs from a G610C mouse model of OI with a Gly610 to Cys substitution in the triple helical region of pro*α*2(I).^[^ ^13^ ^]^ The G610C mutation causes ER disruption and ISR in OBs without ER‐UPR like ATZ in hepatocytes, yet it also causes ER‐UPR in hypertrophic chondrocytes (such as ATZ in monocytes).^[^ ^19^ ^]^ The ISR without ER‐UPR in OBs and with ER‐UPR in hypetrophic chondrocytes were observed even in adjacent cells within the same tissue section.^[^ ^20^ ^]^ While consistent NF*κ*B signaling activation was not detected in G610C OBs, the ATZ‐like cellular response could not be excluded.^[^ ^13^ ^]^ Similarly, no upregulation of BIP and activation of IRE1 and ATF6 was observed in cultured fibroblasts from some OI patients yet such evidence of ER‐UPR was observed in fibroblasts from other patients (akin to ER‐UPR in response to ER disruption by some but not all viral proteins).^[^ ^21^ ^]^ Together, all these observations may indicate both that (a) the ER‐UPR may not be the only pathway of the cellular response to ER disruption by secretory protein misfolding and (b) the ER overload may not be the only alternative to the ER‐UPR.

The latter intriguing possibility and other previous findings in the G610C mouse model stimulated the present analysis of pathways involved in ISR activation in G610C OBs. The G610C mouse mimics a moderately severe mutation found in a large group of OI patients.^[^ ^22^ ^]^ It has become a widely studied animal model of OI.^[^ ^23^ ^]^ Heterozygous (Het) G610C mutation leads to variable bone deformities and occasional (although not common) long bone fractures upon normal daily activities. The deformities and fractures are caused primarily by type I procollagen misfolding in the ER of OBs and resulting OB malfunction.^[^ ^13^, ^24^ ^]^ Homozygous (Hom) mutation causes severe in utero bone deformities and fractures, and Hom animals die at birth.^[^ ^13^, ^22^ ^]^ ER disruption by misfolded procollagen and resulting ISR not accompanied by ER‐UPR were demonstrated both in vivo and in cultured OBs.^[^ ^13^ ^]^ An unexpected pathway of quality control and autophagy of misfolded procollagen was established by live cell imaging.^[^ ^25^ ^]^ However, the pathway of ISR activation remained completely unclear.

The goal of the present study was therefore to answer the following mechanistic questions. First, is ISR activation pathway in G610C OBs different from both ER‐UPR and ER overload? Second, what are the key regulators and transducers of this pathway? To answer these questions, we performed single cell RNA sequencing (scRNASeq) and identified potential markers of this pathway by correlating the cellular response with increasing *Col1a1* transcription in OBs. Since cell isolation for scRNASeq from mouse bones could affect mRNA, we followed up with in situ transcriptomic analysis of fresh‐frozen bone sections with ≈100 µm spatial resolution (srRNASeq) and fluorescent in situ mRNA hybridization (mRNA‐FISH) in fixed bone sections with a single cell resolution. After confirming the cellular response markers, we demonstrated the same response in cultured primary parietal bone OBs by bulk RNASeq and used cultured cells for examining key regulators of this response at the protein level (Figure S1, Supporting Information).

These experiments reveal a consistent picture of robust ISR marked by upregulation of *Hspa9* and *Atf5* (paralogues of *Hspa5* and *Atf4*) yet not accompanied by canonical ER‐UPR, ER overload, or mt‐UPR (in which *Hspa9* and *Atf5* have been implicated^[^ ^26^ ^]^). Taking together the previously published and present evidence, we hypothesize how the ISR might be activated in G610C OBs, and lay the ground for testing our hypotheses in subsequent studies.

## 2 Results

### 2.1 Osteoblast Transcriptome Analysis by scRNASeq

For scRNASeq analysis of G610C mouse OBs, we selected embryonic femurs and tibias ≈18.5 days post conception (E18.5) and parietal bones from pups 4–5 days after birth (hereafter denoted as P5). Small size of mineralized spicules and high porosity of these bones reduced the time needed for isolating single cell suspensions to below 30 min, minimizing mRNA degradation and synthesis during the isolation procedure. To study transcripts of genes involved in cellular response to stress, which may be rapidly made and degraded, we could not use longer isolation procedures reported before.^[^ ^27^ ^]^ We observed no noticeable effects of tissue and cell storage on ice before and after the 30 min isolation for up to ≈4 h when processing multiple samples in different experiments. E18.5 tissue analysis was required to examine effects of Hom G610C in addition to Het G610C, since Hom pups die at birth.^[^ ^13^ ^]^

**Figure 1:**
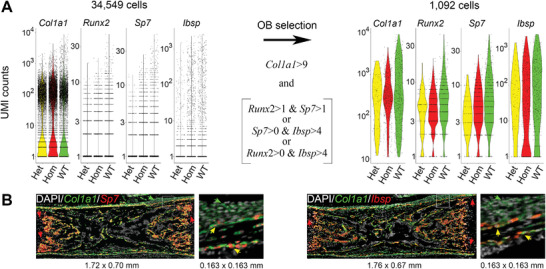
Osteoblast selection in single cell RNA sequencing (scRNASeq) based on marker genes. A) Left: individual mRNA counts based on unique molecular identifiers (UMI) for *Col1a1*, *Runx2*, *Sp7*, and *Ibsp* in cell isolates from femurs and tibias of E18.5 embryos (3 Hom, 511 osteoblasts (OBs); 3 Het, 96 OBs; 4 WT), 485 OBs). Right: UMIs in OBs selected based on the indicated threshold values (optimized as described in Experimental Section). *Runx2* and *Sp7* transcripts are not detected by scRNASeq in some OBs expressing these genes because of their relatively low abundance. *Ibsp* is also not detected in some OBs because of its highly variable expression dependent on OB differentiation. B) Validation of OB marker gene selection by mRNA‐FISH in 5 µm paraffin sections of E18.5 femurs. Co‐expression of *Col1a1* and *Sp7* and/or *Col1a1* and *Ibsp* is observed in all OBs (bone surface cells producing collagen I) and no other cells. White boxes indicate zoomed areas. Yellow arrows point to osteoblasts expressing *Col1a1* and Sp7 (left) or *Col1a1* and *Ibsp* (right). Green arrows point to periosteal fibroblasts expressing only *Col1a1*. Red arrows point to chondrocytes expressing only *Sp7* or *Ibsp*.

We identified OBs based on expression of *Col1a1*, *Runx2*, S*p7*, and *Ibsp* rather than by unsupervised clustering since we did not need to characterize the entire population of isolated cells (**Figure** **1**A). The unsupervised clustering is useful for identifying groups of cells that are not known a priori, but its multiple challenges and ad hoc approximations make it less well‐suited for isolating subsets of cells for which mRNA markers are well‐known.^[^ ^28^ ^]^ Indeed, in unsupervised cell clustering, we observed multiple non‐osteoblastic cells within UMAP or tSNE OB clusters and multiple OBs outside these clusters (Figure S2A, Supporting Information). We validated our more direct approach by mRNA‐FISH of E18.5 femur sections. We confirmed that OBs were distinctly marked by co‐expression of *Col1a1* and *Sp7* or co‐expression *Col1a1* and *Ibsp* even without *Runx2*‐based selection (Figure 1B). We tuned the values of the OB selection thresholds based on the efficiency of excluding cells expressing fibroblast (*Clec3b*), endothelial (*Pecam1*), chondrocyte (*Acan*), smooth muscle (*Acta2*), macrophage (*Cd68*), and neutrophil/macrophage (*Cd33*) marker genes (Figure S2B, Supporting Information).

### 2.2 Cell Stress and OB Malfunction

**Figure 2:**
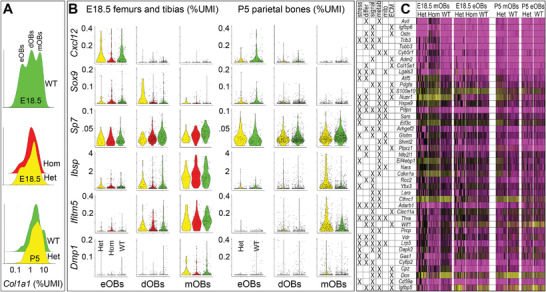
Transcriptomic signatures of osteoblasts (OB) differentiation and function. A) Histograms of *Col1a1* expression in E18.5 long bones (top 2 panels) and P5 parietal bones (bottom panel). Hereafter mRNA expression is reported as percent of the total transcripts in the cell (%UMI). Based on distinct peaks in the *Col1a1* histogram fromWT E18.5 bones, we separated three subpopulations of OBs with *Col1a1* < 0.75%UMI, 0.75 ≤ *Col1a1*≤3.5%UMI, and *Col1a1* > 3.5%UMI in all genotypes and tissues. B) Expression of osteoblast differentiation markers. Based on relative expression of *Cxcl12*, *Sox9*, *Sp7*, *Ibsp*, *Ifitm5*, and *Dmp1*, we classified the first subpopulation as early (eOBs), the second one as differentiating (dOBs), and the third one as mature (mOBs) osteoblasts. C) Heat maps of genes significantly affected by the G610C mutation in mOBs from both E18.5 long bones (Figure 1) and P5 parietal bones (4 Het, 954 OBs; 4 WT, 1521 OBs). The color change from magenta to black to yellow corresponds to increasing gene expression. All genes satisfying the following criteria are shown: i) statistically significant effect of the mutation both in Hom E18.5 mOBs and Het P5 mOBs (*p* < 0.05, Wilcoxon test with Bonferroni correction); ii) at least twofold change in Hom versus WT E18.5 mOBs; iii) reproducible effect of the mutation in two independent experiments with P5 OBs; and iv) involvement of the gene in at least two processes essential for OB function (marked by X). The following essential processes annotated in the Gene Ontology (GO) database were chosen for the analysis: cell death, stress, transcription, translation (stress); cell differentiation (differ); cell signaling (signal); metabolism (metab); mitochondrial function (mito); and extracellular matrix (ECM) synthesis/function (ECM). Cautionary notes: a) The Bonferroni correction for multiple comparisons may prevent detection of some genes affected by the mutation (particularly when low expression limits accurate mRNA quantification by scRNASeq). b) While useful for initial bioinformatic analysis, GO annotations may be misleading since they are incomplete and do not require validating experiments.

To examine how the G610C mutation affected OB differentiation and function, we further subdivided OBs into subpopulations with low, mid‐range, and high expression of *Col1a1*. In wild type (WT) E18.5 long bones, these subpopulations could be separated based on distinct peaks in the *Col1a1* expression histogram (**Figure** **2**A, top panel). Expression of OB differentiation markers suggested that these subpopulations represented early (eOBs), differentiating (dOBs), and mature OBs (mOBs), respectively (Figure 2B). The same subpopulations appeared to be present in Het and Hom E18.5 OBs as well as in WT and Het P5 OBs, although histograms of P5 OBs had additional structure (Figure 2A,B). Importantly, the fraction of mOBs was reduced in Het and further reduced in Hom animals, indicating deficient OB maturation (Figure 2A,B) consistent with our earlier findings.^[^ ^13^ ^]^

In addition to suppressed progression to mOBs, scRNASeq revealed clear evidence of significant changes in mOB function. In particular, we observed significant changes in the expression of genes regulating cell signaling (e.g., Wnt and IGF pathways crucial for OBs), cell cycle, migration, metabolism, and ECM synthesis (Figure 2C; Figure S3, Supporting Information). Most of these genes were affected in mOBs but not in eOBs, suggesting that they were responding to the increased synthesis of mutant collagen.

**Figure 3:**
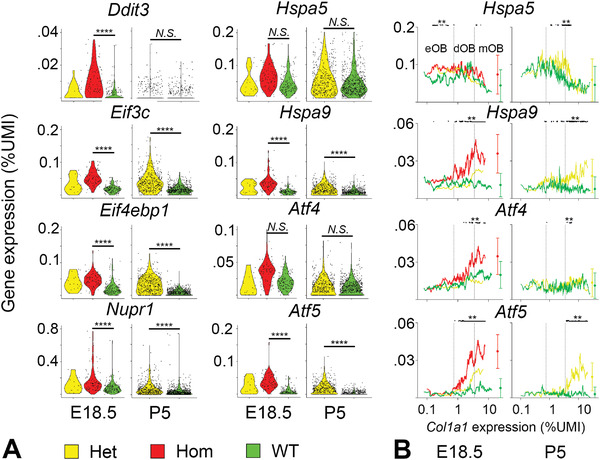
Expression of UPR and ISR genes. A) Violin plots of *Ddit3, Eif3c, Eif4ebp1, Nupr1, Hspa5, Hspa9, Atf4*, and *Atf5* expression in mature osteoblasts (mOBs). More than 20% change in gene expression with *p* < 0.05 (Wilcoxon test with Bonferroni correction) was considered significant. N.S. = not significant, ^****^ *p* < 0.0001. B) Dependence of *Hspa5*, *Hspa9*, *Atf4*, and *Atf5* expression on *Col1a1* transcription (running average over 20 cells sorted in the order of increasing *Col1a1*). Small black dots on top mark mRNA upregulation in Hom versus WT (E18.5) and Het versus WT (P5) with estimated significance of at least ^**^ *p* < 0.01. Circles with error bars show mean transcription in mOBs and mean value of the standard deviation for the running average across the full range of *Col1a1*.

Over 20% of genes, we identified as significantly up‐ or downregulated both in E18.5 and P5 mOBs (>2‐fold in Hom versus WT) were previously annotated in the Gene Ontology (GO) database as involved in cell survival/death, transcription, and/or translation (Figure 2C). Among these were ISR regulators *Ddit3*, *Eif3c*, *Eif4ebp1*, *Nupr1*, and *Trib3* (Figure 2C, **Figure** **3**A; Figure S4, Supporting Information). *Eif4ebp1*, *Eif3c*, *Nupr1*, and *Trib3* were significantly upregulated in G610C mOBs from both E18.5 and P5 bones. *Ddit3* was also upregulated in all G610C mOBs, but this upregulation reached statistical significance only in Hom E18.5 mOBs. Low overall expression of *Ddit3* prevented conclusive statistical analysis of more subtle changes in Het mOBs.

The ISR was activated by upstream events other than the ER‐UPR or ER overload, consistent with our previous findings.^[^ ^13^ ^]^ We observed no upregulation of *Hspa5* either in Het E18.5 or Het P5 mOBs, in contrast to what would be expected in the ER‐UPR (Figure 3). In Hom E18.5 mOBs, *Hspa5* expression was higher than in Het or WT, but the difference was not significant after the Bonferroni correction (Figure 3A). In any case, this upregulation was unrelated to procollagen misfolding inside the cell since increased misfolding expected at high *Col1a1* transcription reduced both the differential and absolute expression of *Hspa5* (Figure 3B). We found no changes in mRNA of any other ER‐UPR genes in either E18.5 or P5 mOBs at high *Col1a1* transcription (Figure S4, Supporting Information). Although we observed altered expression of many genes involved in cell signaling, none of these genes were key regulators or targets of the NF*κ*B pathway reported to be involved in the ER overload (Figure 2C; Figure S3, Supporting Information).

Interestingly, *Creb3l1* was upregulated in Het P5 but not in more severely affected Hom E18.5 OBs (Figure S4, Supporting Information). This gene encodes an ATF6‐like ER membrane stress receptor regulating *Col1a1*,^[^ ^29^ ^]^ yet it does not appear to be essential for ISR activation (Figure S4, Supporting Information).

It is important to note that the canonical ISR transducer *Atf4* was slightly upregulated in Hom E18.5 versus WT E18.5 but not in Het E18.5 or Het P5 OBs (Figure 3A). However, *Atf4* may be directly involved in OB differentiation.^[^ ^30^ ^]^ OBs may thereby regulate *Atf4* expression differently from other cells, and interpretation of the observed small difference between Hom and WT cells may not be straightforward.

### 2.3 Upregulation of Hspa9 and Atf5

The best clue to a potential mechanism of this unusual cellular response was provided by an unexpected upregulation of *Hspa9* and *Atf5* transcription in Hom and Het OBs (Figure 3A,B). *Hspa9* and *Atf5* are paralogues of *Hspa5* and *Atf4*, respectively. An HSP70 family chaperone encoded by *Hspa9* (mt‐HSP70/GRP75) is a mitochondrial paralogue of BIP, which is synthesized in the cytosol and translocated to mitochondria, where it assists protein folding like BIP does in the ER.^[^ ^31^ ^]^ ATF5 and ATF4 proteins are believed to have overlapping functions in the ISR, although ATF5 has not been as extensively studied.^[^ ^5^ ^]^

We confirmed activation of mt‐HSP70 and ATF5 by examining the dependence of their transcription on that of *Col1a1* (Figure 3B). Upregulation of *Hspa9* and *Atf5* correlated with *Col1a1* transcription in Hom and Het OBs and occurred only above a threshold level of *Col1a1*. No change in *Hspa9* and *Atf5* mRNA was observed with increasing *Col1a1* transcription in WT OBs. The increases in *Hspa9* and *Atf5* were more pronounced in Hom compared to Het cells, also consistent with increased procollagen misfolding. In general, *Hspa9* and *Atf5* expression in different types of cells correlated with collagen‐specific chaperone *Serpinh1*/HSP47 (Figure S5, Supporting Information), suggesting the importance of mt‐HSP70 and ATF5 for collagen‐producing cells.

The evolutionary origin and similar function of mt‐HSP70 and ATF5 to the master regulators of the ER‐UPR and ISR suggested that we must understand their role, but the OB transcriptome did not match known pathways of cellular response to stress involving these proteins. Previously, *Hspa9* and *Atf5* were reported to be activated by misfolding of mitochondrial proteins.^[^ ^26^ ^]^ We observed changes in multiple genes encoding mitochondrial proteins or otherwise known to indicate mitochondrial distress (Figure 2C). At the same time, we did not observe other key features of the mt‐UPR such as upregulation of *Hspd1*, *Hspe1*, *Dnaja3*, *Clpp*, and *Lonp1* with increasing *Col1a1* (Figure S6, Supporting Information), even though LONP1 was reported as a chaperone partner of mt‐HSP70.^[^ ^32^ ^]^ Therefore, we examined whether the *Hspa9* and *Atf5* upregulation and other key transcriptomic changes we observed were a true response to procollagen misfolding rather than just an effect of cell isolation for scRNASeq on Hom and Het OBs. While the latter would still indicate altered cellular homeostasis in Hom and Het OBs, it could be unrelated to physiologically important cellular response pathways.

### 2.4 srRNASeq in Frozen Tissue Sections

**Figure 4:**
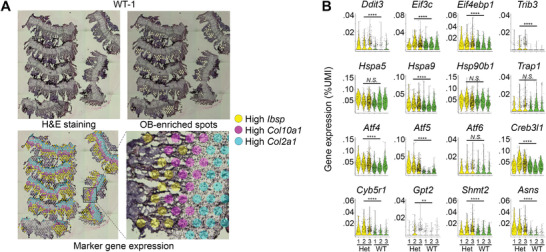
Transcriptomic analysis of osteoblast enriched spot in frozen tissue sections. A) Mapping of OB‐enriched spots for transcriptomic analysis based on marker gene expression. Top left: Frozen sections of growth plate and adjacent trabecular bone from proximal tibia of a wild type mouse (WT‐1 sample). Top right: Osteoblast‐enriched spots (yellow circles) selected for transcriptomic analysis based on above‐threshold expression of *Col1a1*, *Runx2*, *Sp7*, and *Ibsp* (similar to Figure 1) as well as below‐threshold expression of *Col2a1* (<0.15% UMI) and *Col10a1* (<0.03% UMI). Bottom left: Spots with high *Ibsp* (yellow), *Col10a1* (magenta), and *Col2a1* (cyan) expression. Bottom right: zoomed area of the left image illustrating *Ibsp* expression in trabecular area, *Col10a1* expression in the hypertrophic zone and adjacent structures, and *Col2a1* expression in the rest of the growth plate. B) Expression of ISR genes (top row), endoplasmic reticulum (ER) and mitochondrial HSP70 and HSP90 chaperones (second row), transcription factors regulating cellular response to stress (third row), and genes indicating mitochondrial distress (bottom row). Violin plots are shown for each of the 3 Het (yellow) and 3 WT (green) animals examined by srRNASeq. BIP encoded by *Hspa5* is ER‐HSP70, *Hspa9* encodes mt‐HSP70, *Hsp90b1* and *Trap1* encode ER‐HSP90 and mt‐HSP90, respectively. *Cyb5r1*, *Gpt2*, and *Shmt2* encode mitochondrial proteins. Asparagine synthase gene (*Asns*) is a known marker of mitochondrial stress response regulated by ATF4.^[^ ^33^ ^]^ More than 20% change in gene expression with *p* < 0.05 (Wilcoxon test) was considered significant. N.S. = not significant, ^**^ *p* < 0.01, ^****^ *p* < 0.0001.

First, we eliminated potential cell isolation effects by a transcriptomic analysis of OB‐enriched regions in fresh‐frozen sections from proximal tibia in 4.5‐week‐old Het and Wt pups (**Figure** **4**, preliminary analysis of the data was partially reported earlier^[^ ^20^ ^]^). For these experiments, the frozen sections were placed on special 10X Genomics Visium slides containing thousands of encoded oligonucleotides within each of thousands of 55 µm, regularly spaced round spots (Figure 4A). The oligonucleotides were selectively hybridized with the 3’ end of mRNA eluted upon tissue permeabilization, enabling scRNASeq‐like mRNA sequencing with individual spots replacing individual cells, to which we refer as srRNASeq. By selecting the spots enriched in OBs based on their localization relative to the trabecular bone and expression of OB marker genes, we were able to perform the same analysis as for scRNASeq, except for plotting gene expression versus *Col1a1*.

Overall, we observed the same differential gene expression as in scRNASeq, including increased expression of ISR genes (*Ddit3*, *Eif3c*, *Eif4ebp1*, and Trib3), *Atf4*, *Hspa9*, *Atf5*, and genes indicating mitochondrial distress (Figure 4B). Like in scRNASeq, we observed no upregulation of *Hspa5* and other genes encoding ER‐UPR proteins such as ATF6, ER‐HSP90/GRP94, and mt‐HSP90/TRAP1 (Figure 4B). More than 50% of genes shown in the heatmaps in Figure 2C were detected as differentially expressed by srRNASeq as well. Because of its 55 µm diameter, each Visium slide spot could capture mRNA from up to ≈10 cells. The contribution from cells other than OBs could thereby dilute and confound less pronounced effects of the mutation. For instance, *Nupr1* upregulation in Het OBs consistent with scRNASeq was detected by srRNASeq, yet it failed to reach significance (probably because of such confounding effects). Nonetheless, we confirmed the key observations of ISR activation without the ER‐UPR, mitochondrial distress, and upregulation of *Atf5* and *Hspa9* in vivo by avoiding cell isolation. The lack of single cell resolution, however, prevented unambiguous attribution of these effects to OBs versus adjacent cells responding to OB signaling.

### 2.5 mRNA Visualization in Fixed Tissue Sections

**Figure 5:**
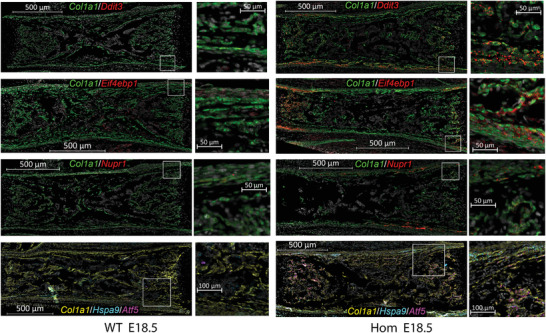
Visualization of *Ddit3*, *Eif4ebp1*, *Nupr1*, *Hspa9*, and *Atf5* mRNA in fixed, paraffin embedded sections of E18.5 femurs. Fluorescent in situ hybridization (FISH) of mRNA in WT and Hom tissue sections was performed at the same time, with the same reagents, and at identical conditions. The slides were imaged at identical fluorescence channel settings and digitally visualized with identical contrast enhancement to preserve relative fluorescence intensity. Cell nuclei labeled with DAPI are shown in grayscale. The other pseudo colors correspond to the gene symbol colors of the corresponding legends. Intense *Col1a1* labeling identifies OBs while weaker *Col1a1* labeling is observed in fibroblasts (mostly within periosteum). White boxes outline regions shown in zoomed panels.

Therefore, we next examined transcription of key ISR genes, *Hspa9*, and *Atf5* in paraffin embedded sections of tissues that were rapidly dissected and fixed to prevent mRNA degradation (**Figure** **5**). The mRNA transcripts were visualized with subcellular resolution by RNAScope mRNA‐FISH assay, which we optimized for bone. This assay involves amplification of the fluorescence signal, which allows mRNA imaging in tissues with high autofluorescence like bone yet precludes accurate mRNA quantification. To better distinguish differences in the expression levels, we thus compared WT and Hom E18.5 femurs.

Consistent with scRNASeq, mRNA‐FISH revealed increased expression of ISR genes, *Hspa9*, and *Atf5* in Hom OBs, which was particularly dramatic within regions of active bone formation adjacent to the growth plates (Figure 5). In mRNA‐FISH images, OBs are identified by intense *Col1a1* labeling as demonstrated in Figure 1B. Because of massive collagen synthesis, *Col1a1* FISH probes label almost entire OB cytoplasm and no autofluorescence background is visible. For much lower expression genes, FISH labeling is mostly punctate, because mRNA is localized at fewer foci. Much lower FISH signal intensity also means that some autofluorescence is visible, but it can be easily distinguished from the punctate FISH signal by its smeared appearance. In WT, most of *Hspa9* mRNA is localized in cells other than OBs. In Hom, the same non‐OB *Hspa9* mRNA is also visible, but much more *Hspa9* mRNA is observed in OBs. Although rigorous statistical analysis of the expression within this assay was impossible, mRNA‐FISH supported the sc‐ and sr‐RNASeq conclusions by showing dramatic upregulation of *Ddit3*, *Eif4ebp1*, *Nupr1*, *Hspa9*, and *Atf5* in most active OBs that were producing bone.

### 2.6 Analysis of Cultured OBs

**Figure 6:**
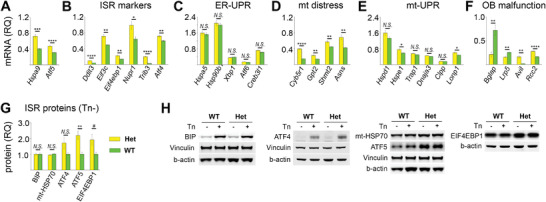
Relative quantity (RQ) of key pathway/function markers in cultured primary OBs. A‐F) RNASeq quantification of mRNA relative to geometric mean of housekeeping genes (*Actg1*, *Actb*, *Mrfap1*, and *Sdha*). G) Cell lysate protein quantification in Het relative to average WT values by Western blotting. H) Representative blots of the proteins and corresponding loading standards (each panel shows a separate blot). Short (3–5 h) treatment with 10 µg mL^−1^ tunicamycin (Tn) was used for validating effects of canonical, acute UPR on BIP and ATF4 (not expected to produce noticeable effects on mt‐HSP70, ATF5, and EIF4EBP1). A) *Hspa9* and *Atf5*. B) ISR genes. C) Endoplasmic reticulum (ER) UPR genes (*Hspa5*, *Hsp90b1*, *Xbp1*, *Atf6*) and *Creb3l1*. D) Markers of mitochondrial distress. E) Markers of mt‐UPR. F) Markers of cellular malfunction: *Bglap* – OB differentiation, *Lrp5* – Wnt signaling, *Avil* – cytoskeleton and cell motility, *Rcc2* – cell cycle. Error bars show standard error of the mean in six replicates (mRNA) and 6–12 replicates (protein). N.S. = not significant, ^*^ *p* < 0.05, ^**^ *p* < 0.01, ^***^ *p* < 0.001, ^****^ *p* < 0.0001. More than 20% (see Table S1, Supporting Information) changes in relative expression with *p* < 0.05 were considered statistically significant. Two‐tailed, heteroscedastic *t*‐test and Mann–Whitney *U*‐test produced consistent significance estimates for most markers, with the only exception of *p* = 0.03 in *t*‐test and *p* = 0.1 in *U*‐test indicated by # (EIF4EBP1 in G). For mRNA analysis, six wells per genotype were examined (3 at 14 and 3 at 21 days after seeding) from two separate cell preparations (two wells per genotype in the first and four wells per genotype in the second experiment). Similarly, six wells per genotype were examined by Western blotting in two separate cell preparations of three wells per genotype. The variation between the experiments was the same as between different wells within the same experiment. Technical variation between different Western blot lanes was comparable to or larger than the variation between different biological samples.

Lastly, we analyzed effects of the G610C mutation on cultured primary OBs from parietal bones of 5‐day old pups, to evaluate whether the cellular response involving *Hspa9* and *Atf5* can be studied in primary cell culture and to examine this response at the protein level (**Figure** **6**). We started from transcriptomic analysis of OB cultures by RNASeq at 8, 14, and 21 days after seeding. The transcription of key genes discussed above at 21 days was similar to 14 days, indicating a steady state of collagen matrix synthesis (Table S1, Supporting Information). At 8 days, the cells just reached confluency and had significantly different transcriptome, e.g., 3–4 times less *Col1a1* mRNA. Consequently, the data from 14 and 21 days were combined and normalized based on expression of four housekeeping genes (*Actg1*, *Actb*, *Mrfap1*, and *Sdha*) selected and validated by scRNASeq as described in Supporting Information (where we also explain the reason behind selecting this approach).

The results of RNASeq were completely consistent with scRNASeq, srRNASeq, and mRNA‐FISH, revealing the same mutation effects, establishing primary OB culture as a valid model, and further confirming our observations. We again observed significant upregulation of *Hspa9*, *Atf5*, and ISR genes (Figure 6A,B) without changes in the ER‐UPR markers *Hspa5*, *Hsp90b1*, *Xbp1*, and *Atf6* (Figure 6C). *Creb3l1* transcription appeared to be increased but was not marked as statistically significant because the observed <20% change would require a much larger number of samples to be statistically validated. We again found large changes in transcription of multiple mitochondrial genes and markers of mitochondrial distress (Figure 6D, c.f. Figure 2C and Figure 4B) yet no clear evidence of the mt‐UPR (Figure 6E, c.f. Figure S6, Supporting Information). Like in vivo, cultured primary OBs showed evidence of malfunction, which included but was not limited to altered differentiation, signaling, motility, and cell cycle (Figure 6F). It must be noted that cells not producing collagen I were also present in the primary culture together with OBs, thereby blunting effects of the mutation.

Finally, we measured expression of *Hspa5* (BIP), *Hspa9* (mt‐HSP70), *Atf4* (ATF4), *Atf5* (ATF5), and the ISR marker *Eif4ebp1* (EIF4EBP1), for which we found reliable antibodies, at the protein level (Figure 6G,H). No difference between BIP in WT and Het cells matched our previous findings^[^ ^13^ ^]^ and the transcriptomic observations. In contrast, acute ER‐UPR caused by pretreatment of both WT and Het cells with 10 µg mL^−1^ tunicamycin for several hours resulted in dramatic increase of BIP in the cell lysate. The increase in ATF4, ATF5, and EIF4EBP1 in Het cells was also completely consistent with the RNASeq observations. Only the ATF5 observation was highly statistically significant (*p* = 0.002), which was not surprising given lower quantitative accuracy and reproducibility of Western blots compared to RNASeq. Unlike *Hspa9* mRNA, we observed no increase in the corresponding mt‐HSP70 protein in Het cells.

## 3 Discussion

To confirm a distinct cellular response to misfolded procollagen accumulation in the ER and identify its markers, we relied on moderate ER disruption^[^ ^13^ ^]^ in OBs from a G610C mouse model of mild‐to‐moderate OI.^[^ ^22^, ^24^ ^]^ In these OBs, the ISR‐induced reduction in protein synthesis and misfolded procollagen autophagy are sufficient for preventing secondary effects of ER homeostasis breakdown, including misfolding of other proteins and subsequent secondary ER‐UPR.^[^ ^20^, ^25^ ^]^ In our opinion, pronounced yet moderate cellular response is the key to reconstructing how misfolded procollagen affects the cell and causes the ISR without confounding secondary effects of the ER homeostasis breakdown downstream of the initial ISR.

Procollagen misfolding and accumulation in the ER, ER disruption, and ISR in G610C OBs were demonstrated before, but neither causality (ER disruption versus extracellular signals) nor the activation pathway of the ISR were understood.^[^ ^13^, ^25^ ^]^ To begin addressing this knowledge gap, we selected transcriptomic analysis as the most versatile approach to identifying relevant cellular pathways in vivo and in culture. This analysis established the causality and distinct features of this response, finally enabling formulation of several data‐based hypotheses for the cellular response pathway for future studies.

As presumed but not demonstrated earlier, the ISR in G610C OBs is activated by accumulation of misfolded procollagen in the ER rather than by extracellular signaling from ECM altered by secreted mutant collagen (or from other cells affected by this ECM). Upregulation of ISR genes only in mature OBs (mOBs) expressing much more *Col1a1* than immature ones (Figure 2A), and exponential‐like response of these genes to increasing *Col1a1* expression validate this causal relationship (see, e.g., *Atf4*, *Atf5*, *Ddit3*, *Eif3c*, and *Eif4ebp1* in Figure 3 and Figure S4, Supporting Information). Interestingly, expression of ER‐UPR genes like *Hspa5* and *Xbp1* provides a contrasting example of an apparent OB stress response to extracellular signals from ECM or other cells. Expression of these genes is altered in immature OBs from Hom tissues (which do not exhibit detectable ISR, yet this effect disappears at higher *Col1a1* expression in mOBs (Figure 3 and Figure S4, Supporting Information).

While caused by ER disruption upon accumulation of misfolded procollagen, the ISR activation in G610C OBs does not involve the ER‐UPR and ER overload pathways or the corresponding protein quality control (QC) systems in the ER lumen. Indeed, the discordant expression of ISR and ER‐UPR genes supports the previous finding of the ISR activation being unrelated to ER‐UPR.^[^ ^13^ ^]^ The lack of NF*κ*B targets among consistently upregulated genes identified by unbiased statistical analysis (Figure 2C) confirms the lack of ER overload. No changes in the expression of *Hspa5*, *Hsp90b1*, other luminal QC chaperones, or the ER‐UPR receptors in mOBs (Figures 3, 4, 6, 4B, 6C; Figure S4, Supporting Information; and earlier study^[^ ^13^ ^]^) indicate that mutant G610C procollagen escapes the QC in ER lumen. This interpretation of the data is consistent with equally efficient secretion of molecules with and without the mutant pro*α*2(I) chain.^[^ ^13^, ^25^ ^]^ Mutant and normal procollagen enter ER exit sites (ERESs) together, where misfolded procollagen aggregates tend to prevent formation of ER‐Golgi transport intermediates and initiate ERES degradation by lysosomes.^[^ ^25a,c^ ^]^ The resulting blockage and depletion of functional ERESs needed for protein export from the ER causes procollagen accumulation in the ER lumen, dilation of the lumen, and therefore ER disruption.^[^ ^25^ ^]^ However, the ER lumen does not seem to have receptors recognizing this disruption. Therefore, the ISR is likely activated by signals from ERESs or other organelles.

The increased transcription of *Hspa9*, *Atf5*, and other genes encoding proteins involved in or responding to mitochondrial function (Figures 3, 4, 5, and 6D) may point to such recognition by mitochondria. *Hspa9* encodes mt‐HSP70, which is a paralogue and member of the same HSP70 family as BIP and which is known to be upregulated in the mt‐UPR like BIP in the ER‐UPR.^[^ ^26^, ^31^, ^34^ ^]^ However, cytosolic and mitochondrial mt‐HSP70 cannot bind to procollagen, which is localized exclusively within the secretory pathway compartments. Hence, it cannot activate the OB response like BIP, which activates the ER‐UPR by binding to misfolded proteins in the ER lumen. *Hspa9* upregulation is also unlikely to be related to bona fide mt‐UPR since it is not accompanied by changes in transcription of other mt‐UPR proteins (Figures 4B and 6E; Figure S6, and Table S1, Supporting Information). Instead, *Hspa9* may be activated by other forms of mitochondrial distress, e.g., by disruption of the mitochondria associated ER membrane (MAM). MAM interacts with mitochondria via mt‐HSP70.^[^ ^31a^ ^]^ MAM also contains high levels of calnexin and calreticulin,^[^ ^31a^ ^]^ which bind procollagen in the ER and may thereby provide a connection between procollagen accumulation in the ER and mt‐HSP70. MAM and/or mitochondria may thus act as the ER disruption sensors and mt‐HSP70 may be involved in the ISR activation.

Upregulation of mt‐HSP70 mRNA (Figures 3, 4, 5, 6) but not the protein (Figure 6G) is consistent with this hypothesis but requires further investigation. For instance, disruption of ER‐mitochondria interactions at MAMs could trigger a distinct mitochondria stress signaling that causes transcriptional activation of *Hspa9* by the ISR yet does not cause an increase in mt‐HSP70. The latter may not be needed for alleviating the MAM disruption, unlike chaperoning mitochondrial protein folding in mt‐UPR. Increased degradation or reduced translation of the mt‐HSP70 in this stress response may then balance the increased transcription of *Hspa9*.

The MAM disruption hypothesis is supported by the increased expression of ATF5 both at the mRNA and protein levels (Figures 3, 4, 5, 6). ATF5 has been proposed to be a mammalian orthologue of *Caenorhabditis*  *elegans* ATFS‐1, which like ATFS‐1 may be prevented from entering mitochondria upon mitochondrial distress, initiating and/or contributing to the ISR.^[^ ^26^, ^34^ ^]^ *Hspa9* is one of the transcriptional targets of ATF5, which could be behind its transcriptional activation.^[^ ^34^ ^]^ While mitochondrial distress caused by the disruption of ER‐mitochondria contacts could trigger ISR reminiscent of mt‐UPR, ATF5 functions have not been sufficiently well understood yet. The role of ATF5 in the ISR therefore needs to be further investigated, but this is beyond the scope of the present study.

Alternatively, as a paralogue of ATF4, ATF5 may be performing an ATF4‐like, mitochondria‐independent function in highly secretory cells like OBs. Indeed, ATF5 has been shown to regulate the ISR downstream of the ER‐UPR triggered by thapsigargin in highly secretory pancreatic *β*‐cells.^[^ ^35^ ^]^ Stronger upregulation of *Atf5* versus *Atf4* in Hom E18.5 OBs (Figure 3B), significant upregulation of *Atf5* but not *Atf4* in Het E18.5 and P5 OBs (Figures 3B and 6B,G), and upregulation of ISR genes in Hom E18.5 and Het E18.5 and P5 OBs (Figure 3B) indicate that ISR in G610C OBs is regulated by ATF5 rather than ATF4, like in pancreatic *β*‐cells. Moreover, high expression of *Atf5* (and *Hspa9*) correlates with high expression of *Serpinh1* (Figure S5, Supporting Information), which encodes a collagen‐specific chaperone HSP47 and is strongly expressed only in highly secretory cells producing large amounts of different collagens.^[^ ^36^ ^]^ Increased transcription of mitochondrial distress markers such as *Hspa9, Cyb5r1, Gpt2, Shmt2*, and *Asns* (Figures 2, 4, 6D) may then be a consequence rather than the cause of *Atf5* upregulation. The mitochondrial changes we observed may thus occur downstream rather than upstream of the ISR. Our data cannot distinguish these interpretations, but they have allowed us to formulate them. This is the crucial first step toward understanding the exact roles of mt‐HSP70 and ATF5 and eventually unraveling the puzzle of the cellular response to procollagen triple helix misfolding.

In conclusion, the present study (a) confirmed ISR activation in G610C OBs without canonical ER‐UPR or ER overload pathways, (b) established that this cellular response is distinguished by *Hspa9* and *Atf5* upregulation, and (c) pointed to disruption of ER‐mitochondria connections as a possible trigger. Because of feedback loops between the ISR and its upstream and downstream events, the question of whether mt‐HSP70 and ATF5 regulate this response, are regulated by it, or both will require further investigation. Still, we now know that understanding the functions of mt‐HSP70 and ATF5 is likely the key to unraveling the mechanism of noncanonical ISR activation in G610C OBs and that the answer may be found in primary cell culture studies. G610C OBs may not represent a universal model of procollagen misfolding, e.g., because of variable effects of other mutations on the ER environment or variable secondary effects of the ER homeostasis disruption in other cells may lead to secondary ER‐UPR or ER overload. However, this only emphasizes the importance of understanding the noncanonical cellular response in G610C OBs, which appears to be distinct and unobscured by the secondary effects. Regardless of how common it may be, this cellular response is unlikely to be specific to the G610C mutation in OBs or even to procollagen misfolding. Nature tends to reuse the tools it develops. To facilitate studies of this response by anyone interested in joining this quest, which is likely to require efforts by multiple laboratories, all transcriptomic data from the present work is publicly shared at https://www.ncbi.nlm.nih.gov/geo/ (accession numbers: GSE210511, GSE210519, GSE210637, GSE210796). All other tools described here and in our other papers are available upon request.

## 4 Experimental Section

### Animals

Het G610C (B6.129(FVB)‐Col1a2tm1Mcbr/J, strain # 0 07248) and C57BL/6J (strain # 000664) mice were purchased from Jackson Laboratory. The G610C animals were maintained on the C57BL/6J background. Stock C57BL/6J animals were reintroduced into the colony every 6–10 generations and all matings were set up exclusively between males and females from different parent cages (to minimize propagation of sporadic mutations). Since Het × Het matings produced litters mostly at E19.5 and all Hom pups died right after birth, WT, Het, and Hom embryos from Het × Het matings were collected for scRNASeq and mRNA‐FISH at E18.5. Animal care and experiments were performed in accordance with protocols approved by the NICHD and University of Maryland School of Medicine ACUCs (Animal Study Protocol #21‐071).

### scRNASeq

Cells for scRNASeq were extracted from E18.5 femurs and tibias as well as from P4‐P5 parietal bones. Tissues were dissected in ice‐cold PBS, minced, transferred into the Eppendorf tubes, and washed with ice‐cold PBS. Cells were released by digestion with 10 mg mL^−1^ bacterial collagenase type 4 (Worthington) at 37 °C for 15 min, with gentle pipetting up and down every 3–4 min using 1 mL tips with cut ends to reduce cell damage. Cell suspension was washed with ice‐cold PBS (calcium and magnesium free) containing 0.04% weight/volume BSA (PBS/BSA), filtered through 40 µm cell strainer, and spun at 300 g for 5 min at 4 °C. Cell pellets were resuspended in 1 mL PBS/BSA, allowed to settle for ≈1 min, transferred into a new tube (top 3/4 of the volume), washed, and re‐pelleted. Cells were counted, diluted to 500–1000 cells µL^−1^, and processed on Chromium Next GEM chips v3.1 (10X Genomics, ≈10000 cells per well). Chromium processing and subsequent library preparation for sequencing were performed according to 10X Genomics protocols. Analysis of library quality on Bioanalyzer 2100 (Agilent), sequencing, and genomic alignment with Cell Ranger software (10X Genomics) were performed by the Molecular Genomics Core facility of NICHD. A total of 4 WT, 3 Het, and 3 Hom E18.5 femur and tibia cell samples from two separate preparations were examined, each sample containing cells from a single embryo. A total of 4 WT and 4 Het P5 parietal bone cell samples were examined, each sample containing cells pooled from up to five animals with the same genotype.

### srRNASeq

This assay was performed to evaluate effects of the G610C mutation and 4‐phenylbutyric acid (4PBA) treatment on OBs and hypertrophic chondrocytes.^[^ ^20^ ^]^ In the present paper, we describe only effects of the mutation on OBs. Briefly, three separate experiments were performed. In each experiment, two WT and two G610C littermates were treated via intraperitoneal injection daily for 10 days starting at 3 weeks after birth with PBS (control) or 0.4 mg 4PBA (Millipore Sigma) dissolved in PBS. The right tibia was rapidly harvested and freshly frozen in OCT (Sakura Finetek). Up to eight cryosections (10 µm thickness) of the proximal growth plate and adjacent trabecular tissue from each of the animals were placed in each of the four capture areas within a Visium slide, fixed with methanol, stained with H&E, imaged, and permeabilized for 45 min to allow for mRNA binding to slide oligonucleotides. Subsequent cDNA synthesis, amplification, and purification were performed as described in the Visium kit for frozen sections (10X Genomics). Library construction, analysis of library quality with Bioanalyzer 2100, sequencing, and genomic alignment with Space Ranger software (10X Genomics) were performed by the Molecular Genomics Core of NICHD. Sequencing results from all three Het samples treated with PBS passed post‐sequencing quality control (QC, see *Data Normalization and Statistical Analysis*) and were used for the present study as the Het group. One WT sample treated with PBS was lost during processing and one did not pass QC. The transcriptome of the third WT/PBS sample was indistinguishable from transcriptomes of two WT/4PBA samples (consistent with no effect of 4PBA on WT animals). Therefore these three WT samples, all of which passed QC, were used as the WT group for the present study. No Het/4PBA samples were used because of previously described effects of 4PBA on hypertrophic Het chondrocytes, which could produce secondary effects on OBs.^[^ ^20^ ^]^

### Bulk RNASeq and Western Blot Analysis of Cultured Cells

Primary OBs were extracted from parietal bones of 4–5 days old pups as previously described.^[^ ^13^ ^]^ The cells were pooled from multiple pups with the same genotype and cultured at 37 °C in *α*MEM + Glutamax (32 571; Gibco) supplemented with 1% Pen‐Strep (Corning), 10% FBS tested for supporting osteoblastic differentiation (Gemini, GemCell, Lot #A83F821) and 100 × 10^−6^ m ascorbic acid 2‐phosphate (Sigma–Aldrich). Media was replaced every 48–72 h, and always 1 day before RNA extraction or collection of cell lysates for Western blotting.

RNA was collected at days 8, 14, and 21 after seeding and purified with a Direct‐zol kit (Zymo Research). RNA QC on Bioanalyzer 2100, sequencing, and genomic alignment were done at the Molecular Genomics Core of NICHD. Two separate experiments were performed with cells from different litters. In the first experiment, cells were plated at 10 000 cells cm^−2^ (one well per genotype for each time point) and cultured in a CO_2_ incubator at atmospheric O_2_. In the second experiment, cells were plated at 4000 cells cm^−2^ (two wells per genotype for each time point), expanded for 4 days (until ≈50% confluent) in a tri‐gas incubator at 5% O_2_, 5% CO_2_, 90% N_2_ (5% O_2_ supports OB proliferation by suppressing their differentiation), and then transferred to the CO_2_ incubator with atmospheric O_2_. In both experiments, the cells were confluent 2 days prior to initial RNA collection (day 8 after seeding).

For Western blotting, cells pooled from multiple pups with the same genotype were plated at 2000 cells cm^−2^, expanded to confluence (5–7 days) in the tri‐gas incubator at 5% O_2_, and further cultured in the CO_2_ incubator with atmospheric O_2_. Cell lysates were collected 12–15 days after seeding following 3–5 h pretreatment with 10 µg mL^−1^ tunicamycin or DMSO (untreated control). Cells were rinsed with PBS and lysed in RIPA buffer containing lithium dodecyl sulfate, 50 × 10^−3^ m dithiothreitol, 1 × 10^−3^ m phenylmethylsulfonyl fluoride, 5 × 10^−3^ m benzamidine, and 10 × 10^−3^ m *N*‐ethylmaleimide. Samples were denatured at 95 °C for 5–10 min, loaded onto 3–8% Tris‐Acetate or 4–12% Bis‐Tris gels and transferred onto a 0.45 µm nitrocellulose membrane. The blots were blocked with 5% BSA buffer, labeled with primary antibodies against BIP (Cell Signaling, Catalog #3177), HSPA9 (ThermoFisher, PA5‐48035), ATF4 (Cell Signaling, 11 815), ATF5 (ThermoFisher MA5‐32365), 4EBP1 (Cell Signaling, 9644), vinculin (Millipore‐Sigma, V284), and *β*‐actin (Abcam, ab8224). After staining with secondary antibodies conjugated to AlexaFluor or DyeLight dyes (Themofisher), images were captured in an FLA9500 fluorescence scanner (Cytiva, former GE HealthCare) and analyzed with ImageQuant TL software supplied with the scanner.

### mRNA‐FISH

Fluorescent in situ hybridization was performed with Advanced Cell Diagnostics (ACD) RNAScope Multiplex Fluorescent V2 kit and mRNA probes for the genes of interest. Dissected E18.5 hind legs were skinned, fixed with formaldehyde in PBS at room temperature (2% for 2–4 h followed by 4% overnight), and embedded in paraffin without demineralization. A total of 5‐µm sections were cut, deparaffinized, treated with ACD custom reagent for bone (to retrieve epitopes and reduce tissue autofluorescence), hybridized with ACD probes, and stained according to the manufacturer's protocol. The nuclei were counterstained with DAPI. The slides were mounted with ProLong Diamond antifade mounting media (ThermoFisher) and imaged on an AxioScan.Z1 slide scanner (Zeiss) with Plan‐apochromat 40×/0.95 objective in four fluorescence channels (DAPI, Cy3, Cy5, and Cy7). The GFP channel was used as autofluorescence control.

### Data Normalization and Statistical Analysis

Genomic alignment, UMI counting, prefiltering, and initial unsupervised clustering of scRNASeq and srRNASeq data were performed with 10X Genomics software (Cell Ranger for scRNASeq and Space Ranger for srRNASeq). Cell clustering was qualitatively visualized in UMAP and tSNE plots in Loupe Browser (10X Genomics). Independent unsupervised clustering and cluster visualization were also performed in Seurat v4 package for R programming language.^[^ ^37^ ^]^ For subsequent quantitative analysis, the UMI counts together with spatial maps (for srRNASeq) were imported into Seurat objects with the Seurat v4 package, which was also used for data management, sorting, QC, and plotting. Because OBs produce much more RNA and require more energy than most other cells, the QC filters were set at minimum 500 features per cell, no maximum features per cell, and less than 10% mitochondrial features (rather than at default Seurat values). The QC thresholds were tuned by examining multiple datasets. Each dataset was additionally examined for expression of *Apaf1*, *Trp53inp1*, and other cell death markers. When increased expression of these markers was detected (one srRNASeq sample), the entire dataset was discarded. To avoid assumptions and nonlinear transformations that could alter the validity of differential gene expression conclusions, the data that passed the QC were normalized as UMI counts per gene divided by UMI counts per cell, using the RC normalization method in Seurat. Analysis of the raw expression data indicated that more common logarithmic or SCTransform data normalization methods implemented in Seurat were inconsistent with OB gene expression patterns (see Supporting Information). The datasets from different experiments and sequencing runs were then merged without additional processing. OBs were separated from other cells based on threshold values for UMI counts of OB marker genes *Col1a1*, *Runx2*, *Sp7*, and *Ibsp* (Figure 1). Average expression of a gene in a group of cells was calculated as the sum of UMI counts for this gene divided by the sum of all UMI counts in the cells (equivalent to assigning a statistical weight to each cell based on its total UMI counts). Wilcoxon ranked sum test implemented in the Seurat package was used for estimating the probability of mean expression values in WT and G610C cells being the same (*p*‐values). Based on the estimated statistical power, only more that 20% changes in the average expression with *p* < 0.05 were considered statistically significant. The Bonferroni correction was applied to account for multiple comparisons when identifying the list of differentially expressed genes (Figures 2C and 3A). Statistical analysis of running average plots in Figure 3B and Figures 3,4,6 (Supporting Information) was based on the standard Wilcoxon test for each running average window. To account for multiple comparisons of these windows, only points with *p* < 0.01 were marked, avoiding false negatives caused by the Bonferroni correction and reducing false positives to ≤5 in E18.5 (≈500 comparisons) and ≤15 (≈1500 comparisons) in P5 samples. R version 4.1.2 (The R Foundation for Statistical Computing) was used for all computations.

Genomic alignment and QC of bulk RNASeq data was performed by the Molecular Genomics Core facility of NICHD. Because mRNA counts were not based on UMI, direct quantification of % transcripts (like %UMI in sc‐ and srRNASeq) was not possible. Analysis of the raw expression data indicated that FPKM, TPM, DESeq2, or other data normalization methods common in bulk RNASeq normalization could not be used (Supporting Information). Therefore, we had to rely on the old‐fashioned yet robust housekeeping gene normalization method analogous to ΔΔ*C*_T_ approach in quantitative PCR. Validation of the housekeeping genes and the normalization procedure are described in Supporting Information. Statistical significance of the results was evaluated by a two‐tailed Student's *t*‐test and nonparametric Mann–Whitney *U*‐test since some of the data failed the Shapiro–Wilkinson normality test. The statistical analysis was performed in SigmaPlot 13 (Systat Software).

Integral intensities of secondary antibody fluorescence at BIP, mt‐HSP70, ATF4, and ATF5 Western blot bands were normalized by the intensity of large (vinculin) and small (*β*‐actin) molecular weight loading controls in the same gel lane. A single, *β*‐actin loading control was used for the small molecular weight EIF4EBP1. Statistical significance of the difference between mean WT and Het values was evaluated by the *t*‐ and *U*‐ tests similar to the analysis of bulk RNASeq data.

## Conflict of Interest

The authors declare no conflict of interest.

## Supporting information

Supporting Information

## Data Availability

All data not contained within the main text or supporting information are available in a public NCBI GEO database, https://www.ncbi.nlm.nih.gov/geo/ (accession numbers: GSE210511, GSE210519, GSE210637, GSE210796).
