# Supplementary material for: ER, Mitochondria, and ISR Regulation by mt‐HSP70 and ATF5 upon Procollagen Misfolding in Osteoblasts
Source: Adv Sci (Weinh). 2022 Aug 21;9(29):2201273. doi: 10.1002/advs.202201273 (PMC9561870; doi:10.1002/advs.202201273)
Supplement: Supplementary file 1 — Supporting Information [file ADVS-9-2201273-s001.pdf]

Supplementary Table 1. Average relative expression of key genes after 8, 14, and 21 days in primary OB culture (c.f. Figure 6). Among over 50 examined genes, the dispersion of 3 replicates for each time point varied from 3 to 20%. We therefore estimated the measurement error at the upper limit of this variation as ~20%. Most genes listed in this table exhibited much larger than 20% change between 8 and 14 days in Het, WT, or both cultures. In contrast, very few differences between the expression at 14 and 21 days that exceeded 20% could be attributed to random outliers within normal distribution. Therefore, we assumed the cells to be in a steady state of collagen matrix synthesis between 14 and 21 days and pooled the corresponding expression data together.

Gene Symbol	8 d, Het	14 d, Het	21 d, Het	8 d, WT	14 d, WT	21 d, WT
Asns	0.11	0.39	0.29	0.09	0.21	0.21
Atf4	0.28	0.76	0.68	0.30	0.57	0.63
Atf5	0.15	0.46	0.48	0.11	0.29	0.34
Atf6	0.082	0.14	0.14	0.09	0.11	0.13
Avil	0.03	0.20	0.12	0.04	0.02	0.01
Bglap	0.001	0.20	0.22	0.003	0.50	0.94
Clpp	0.04	0.05	0.04	0.04	0.04	0.04
Col1a1	34	106	123	43	156	159
Creb3l1	0.17	0.71	0.73	0.22	0.65	0.61
Cyb5r1	0.09	0.20	0.19	0.07	0.07	0.07
Ddit3	0.02	0.10	0.09	0.02	0.04	0.04
Dnaja3	0.08	0.12	0.11	0.09	0.11	0.10
Eif3c	0.41	0.77	0.66	0.40	0.49	0.50
Eif4ebp1	0.09	0.25	0.20	0.07	0.14	0.13
Gpt2	0.04	0.13	0.10	0.04	0.07	0.07
Hsp90b1	2.1	2.4	1.8	2.2	2.2	1.8
Hspa5	1.5	1.8	1.4	1.7	1.7	1.3
Hspa9	0.41	0.80	0.64	0.41	0.43	0.38
Hspd1	0.47	0.46	0.34	0.51	0.38	0.29
Hspe1	0.20	0.22	0.16	0.20	0.16	0.13
Lonp1	0.09	0.16	0.14	0.10	0.12	0.12
Lrp5	0.08	0.16	0.16	0.07	0.22	0.29
Nupr1	0.05	0.93	1.1	0.05	0.51	0.77
Rcc2	0.24	0.29	0.26	0.24	0.18	0.14
Shmt2	0.15	0.31	0.26	0.15	0.24	0.21
Trap1	0.14	0.15	0.13	0.14	0.13	0.12
Trib3	0.008	0.21	0.19	0.005	0.02	0.03
Xbp1	0.20	0.38	0.34	0.24	0.39	0.37

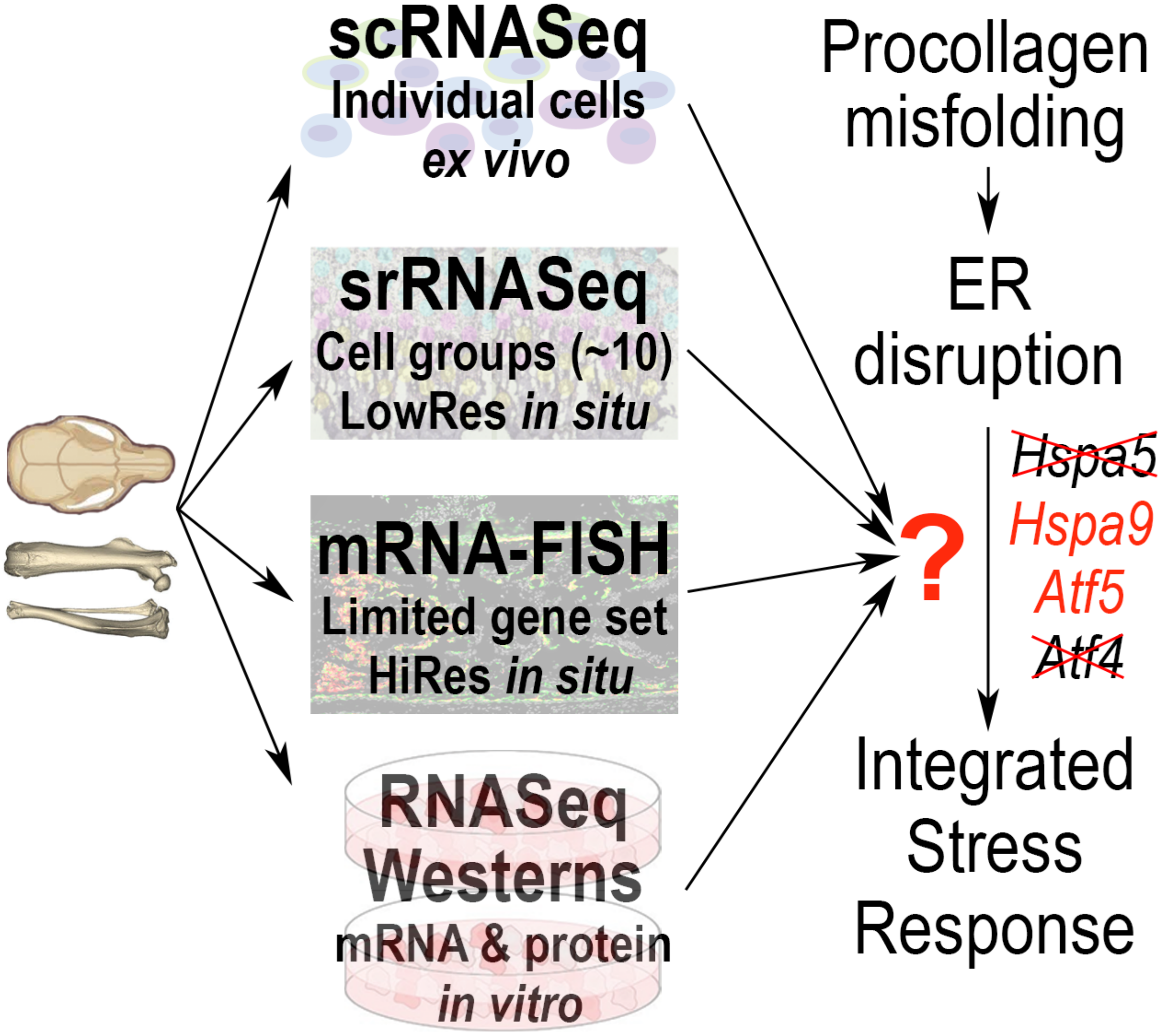

Supplementary Figure 1. Identification of ISR activation pathway(s) in G610C mouse OBs; schematic study layout. 1. Candidates are identified by scRNASeq as ISR-related pathways upregulated by increased synthesis of type I procollagen in G610C OBs isolated from mouse femurs and parietal bones. 2. Potential effects (artifacts) of cell isolation for scRNASeq are eliminated by validating the findings with scRNASeq-like sequencing of mRNA from thousands of regularly spaced 55 μm spots in fresh frozen tibia sections (srRNASeq). 3. Upregulation of key marker genes is further validated with subcellular resolution by mRNA-FISH in fixed femur and tibia sections. 4. The corresponding key proteins are analyzed by Western blotting in primary OB cultures after validating the cell culture approach by comparing bulk RNASeq of cultured OBs with sc- and sr-RNASeq.

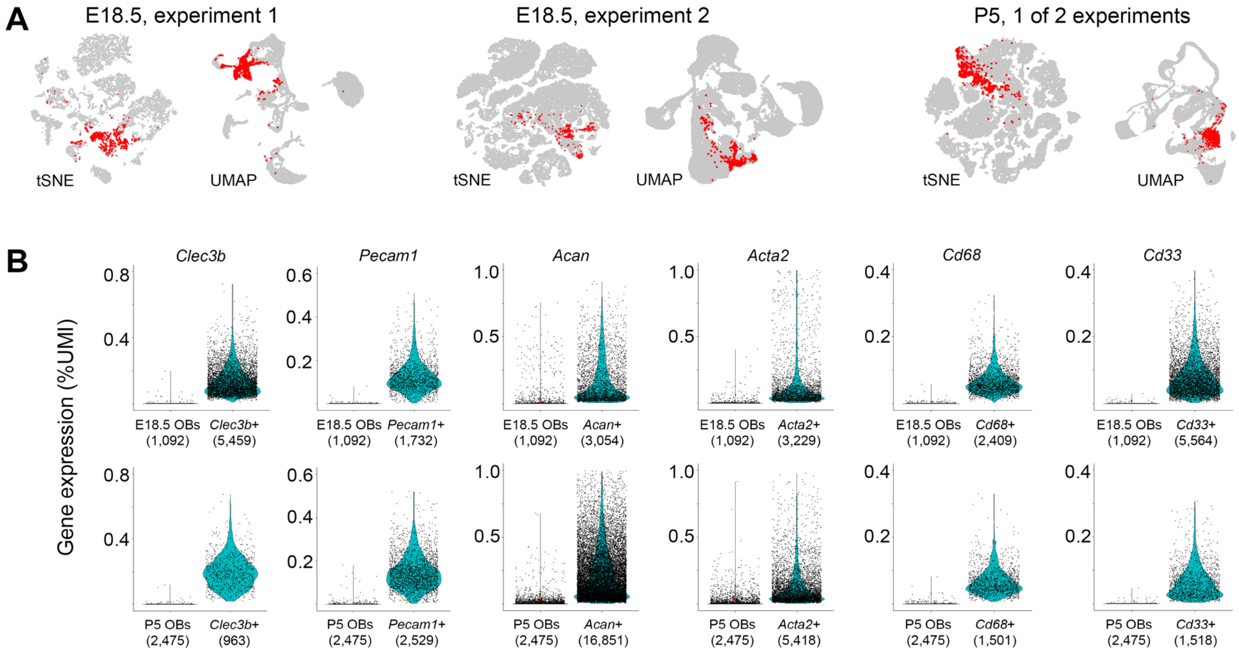

Supplementary Figure 2. OB identification in scRNASeq data. **A.** Localization of OBs (defined as shown in Fig. 1) on tSNE and UMAP plots. Two independent E18.5 cell preparations (1 WT and 1 Hom in experiment 1; 3 WT, 3 Het, and 2 Hom in experiment 2) and one of two P5 cell preparations (2 WT and 2 Het) are shown. PCA analysis was performed with default settings within Cell Ranger program from the assay manufacturer (10X Genomics). OBs were found to form multiple tight clusters plus a scattered pattern of isolated cells. Similar patterns were observed when OBs were selected based on coexpression of *Col1a1*/*Runx2*/*Sp7*/*Ibsp*, expression of *Bglap*/*Bglap2*, or expression of *Ifitm5*. OB clustering was not improved by PCA performed with Seurat version 4.[1] Multiple cells not expressing key OB marker genes were found within tight OB clusters. **B.** Expression of fibroblast (*Clec3b*), endothelial (*Pecam1*), chondrocyte (*Acan*), smooth muscle (*Acta2*), macrophage (*Cd68*), and neutrophil/macrophage (*Cd33*) marker genes in OBs and the corresponding cell subpopulations from E18.5 (top row) and P5 (bottom row) cell preparations. The number of cells in each subpopulation is shown in parentheses. The OB selection thresholds for *Col1a1*, *Runx2*, *Sp7*, and *Ibsp* (Fig. 1) were tuned to simultaneously maximize the separation between the cells and the number of OBs at each differentiation stage. Importantly, some expression of *Acan*, *Acta2*, *Clec3b*, *Pecam1*, and to a much smaller extent *Cd33*/*Cd68* in OBs may be expected (particularly at early differentiation stages).

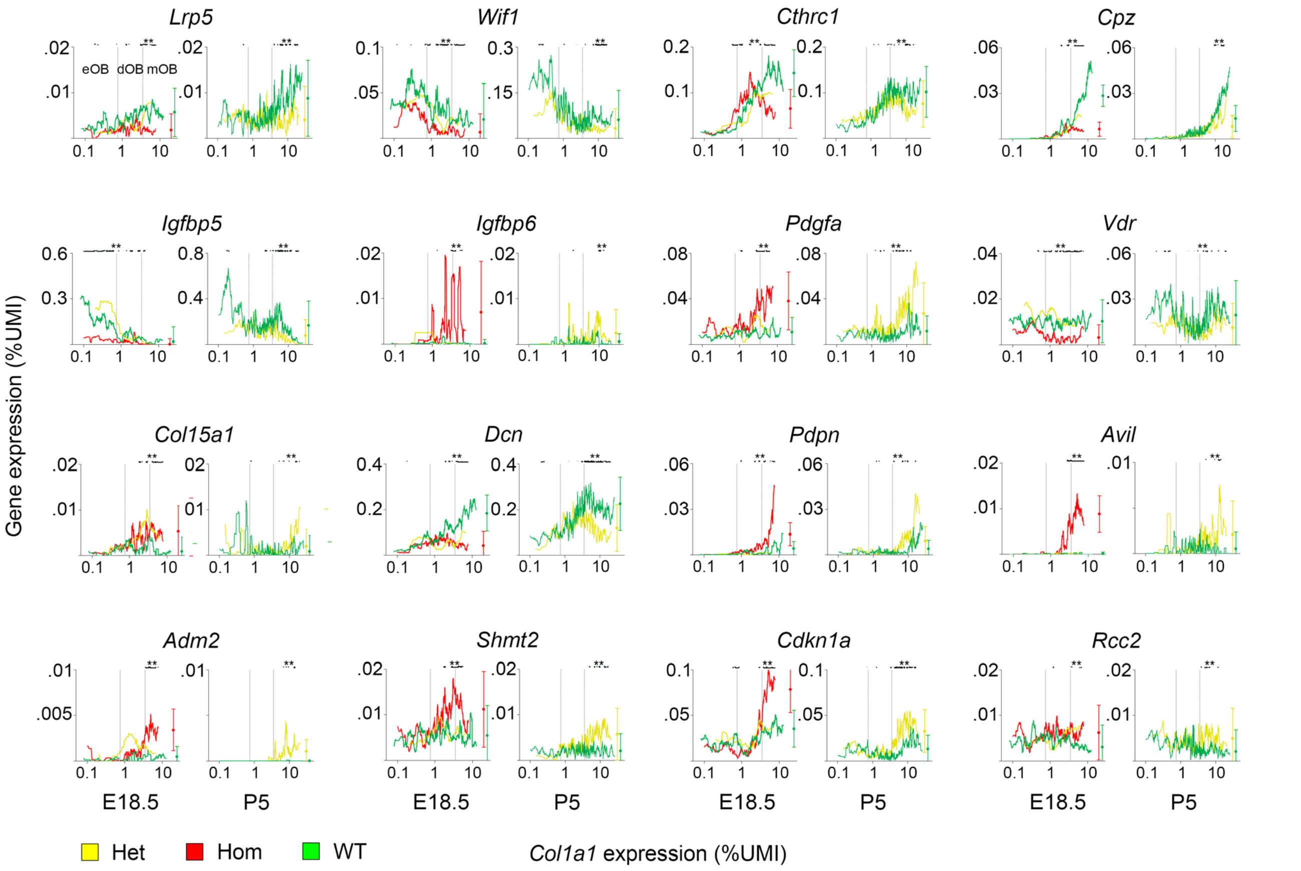

Supplementary Figure 3. OB malfunction. The plots show correlations between expression of selected genes and *Col1a1* transcription in E18.5 and P5 OBs. They represent 20-point running average for cells sorted in the order of increasing *Col1a1* expression. The vertical dotted lines separate subpopulations of early (eOB), differentiating (dOB), and mature (mOB) osteoblasts based on *Col1a1* transcription as described in Fig. 2. Small black dots on top of the plots mark significantly different gene expression ($p <$ 0.01, **) in Hom vs. WT (E18.5) and Het vs. WT (P5). The significance was estimated by the Wilcoxon test for each pair of running average windows centered at the same or closely matching *Col1a1* expression. Circles with error bars show mean transcription in mOBs and mean value of the standard deviation (SD) for the running average across the full range of *Col1a1*.

The G610C mutation clearly affects Wnt (top row), IGF (*Igfbp5*/*6*), and other (*Pdgfa*, *Vdr*) signaling pathways crucial for OB differentiation and function. ECM synthesis (*Col15a1*, *Dcn*), cell migration (*Pdpn*, *Avil*), cell metabolism (*Adm2*, *Shmt2*), and cell cycle (*Cdkn1a*, *Rcc2*) are also significantly altered. The latter effects of the G610C mutation appear only at high collagen expression and coincide with upregulation of ISR markers (Fig. 3b), suggesting that they are caused by procollagen misfolding inside the cell rather than by signals from outside (ECM and other cells). Changes in the expression of *Cthrc1* and *Pdgfa* seem to have the same etiology. In contrast, effects of the mutation on *Wif1* and *Vdr* appear to be independent of the osteoblast differentiation stage and level of *Col1a1* transcription, suggesting that they are caused by altered cellular environment rather than cell-intrinsic factors. The assay sensitivity is not sufficient for similarly distinguishing the etiology of changes in *Lrp5*, *Cpz*, and *Igfbp5*/*6*.

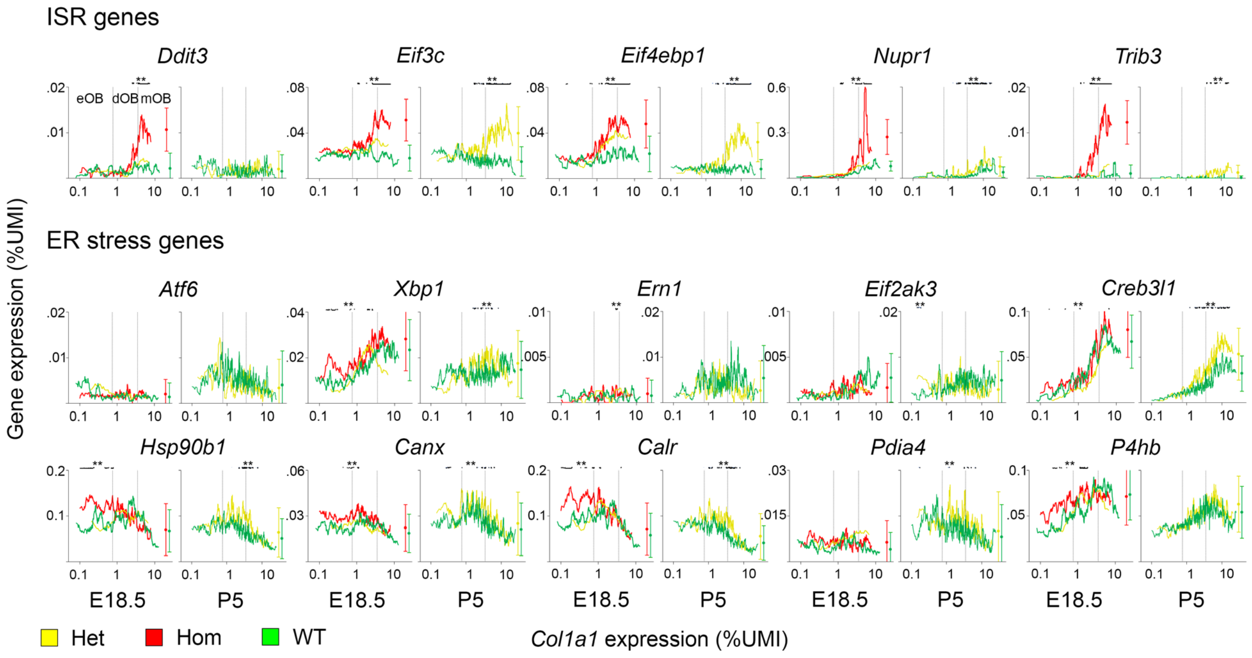

Supplementary Figure 4. Effects of collagen expression on ISR and UPR genes. These plots complement Fig. 3B by showing how transcription of additional ISR, UPR, and *Creb3l1* genes depends on *Col1a1* in E18.5 and P5 OBs (20-point running average). Small black dots on top indicate significant mRNA upregulation in Hom vs. WT (E18.5) and Het vs. WT (P5), unlike Supp. Fig. 3 where they show both up- and down-regulation of the genes. Like in Supp. Fig. 3, circles with errors bars show mean transcription in mOBs and mean value of the SD for the running average across the full range of *Col1a1*.

Strong upregulation of *Atf5* (Fig. 3B), *Ddit3*, *Eif3c*, *Eif4ebp1*, *Nupr1*, and *Trib3* at high *Col1a1* expression unequivocally demonstrates ISR activation by misfolding of G610C procollagen. Minimal change (≤ 1 SD) or downregulation of *Hspa5* (Fig. 3B) and other UPR genes with increasing *Col1a1* is consistent with the lack of UPR upstream of ISR in G610C osteoblasts, which we reported before.[2] Transcription of *Hspa5* and other general ER chaperones (*Hsp90b1*, *Canx*, *Calr*, *Pdia4*, *P4hb*) is upregulated by all UPR pathways.[3] Increased transcription of *Atf6*, *Xbp1*, *Ern1* (IRE1), and *Eif2ak3* (PERK) has been reported in UPR as well.[4] Increased *Atf6* transcription may maintain cellular homeostasis when ATF6 is translocated to Golgi and cleaved. Increased *Xbp1* transcription may be a similar response to its alternative splicing. However, upregulation of *Ern1* and *Eif2ak3* may not be a common feature of the corresponding IRE1 and PERK pathways, although reported in HeLa cells treated with thapsigargin.[4] IRE1 and PERK functions do not involve cleavage or degradation of these proteins.

Increased transcription of *Creb3l1* in Het P5 OBs occurs in the same range of *Col1a1* expression as ISR activation, but no effect of the mutation on *Creb3l1* in Het E18.5 and Hom E18.5 OBs indicates that CREB3L1 is not essential for activating ISR in G610C OBs. *Creb3l1* encodes an ATF6-like ER membrane sensor of ER disruption (CREB3L1/OASIS). Nearly perfect correlation with *Col1a1* points to its transcriptional regulation. It is one of OI genes, which is known to regulate *Col1a1*.[5] CREB3L1 is cleaved like ATF6 upon ER disruption,[5] yet *Creb3l1* transcription is not affected by the G610C mutation in E18.5 OBs despite particularly severe ER disruption and ISR in Hom cells.

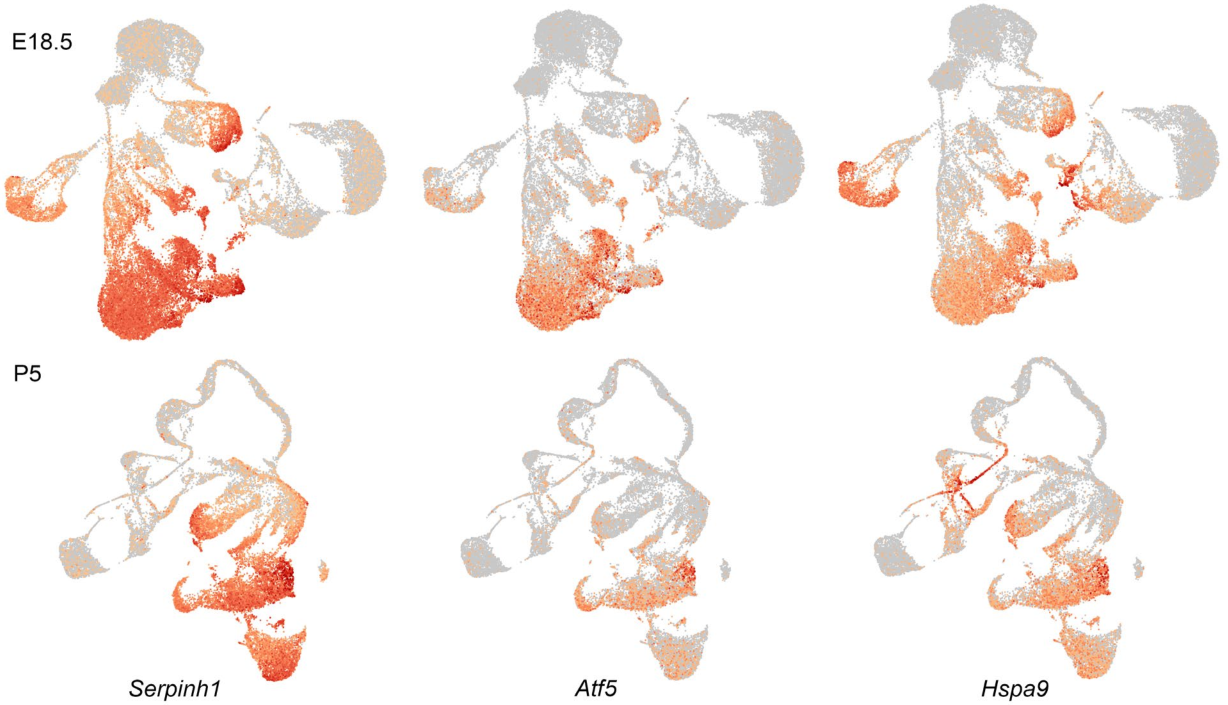

Supplementary Figure 5. Correlation between expression of *Serpinh1*, *Atf5*, and *Hspa9* in different cells. UMAP plots of all cells extracted from E18.5 femurs and tibia (top row) as well as P5 parietal bones (bottom row) are shown. Each dot represents an individual cell. The intensity of the red is proportional to logarithm of the UMI count. *Serpinh1* encodes an HSP47 chaperone, transcription of which parallels that of collagens.[6] Therefore, *Serpinh1* transcription reveals most collagen-producing cells in bone and surrounding tissues.

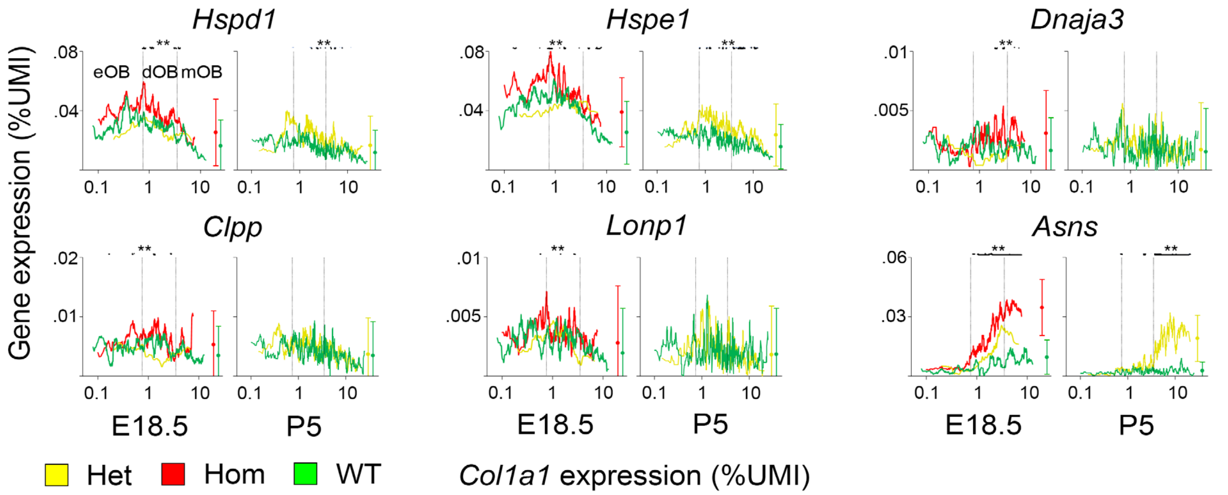

Supplementary Figure 6. Expression of key mitochondrial UPR (mt-UPR) genes. Dependence of the expression of genes known to be transcriptionally upregulated in canonical mt-UPR on the expression of *Col1a1* (for *Hspa9* and *Atf5* see Fig. 3B). Similar to Fig. 3B and Supp. Fig. 4, the curves show 20-point running averages, black dots show gene upregulation with $p < 0.01$ within each running average window, and circles with error bars show mean expression in mOBs and standard deviation averaged over the full range of *Col1a1*. Only *Asns* increases with *Col1a1*, is highly upregulated in mOBs, and is therefore affected by the ER disruption upon G610C procollagen misfolding. Much smaller upregulation of *Hspd1* (mt-HSP60) and *Hspe1* (mt-HSP10) is independent of *Col1a1* expression and therefore likely associated with extracellular effects of the secreted mutant procollagen that alters the ECM and cell-ECM interactions. *Dnaja3* (mt-DNAJ) and genes encoding mt-UPR proteases (*Clpp* and *Lonp1*) do not appear to be upregulated. The few groups of cells with $p < 0.01$ in Hom E18.5 (5-10 *Col1a1* spots) are likely stochastic outliers since ~ 5 outlier spots are expected due to multiple comparison effects on the statistical analysis (see Methods). However, low expression of the latter genes precluded their accurate quantification by scRNASeq (no UMI counts in many OBs). Overall, the observed mitochondrial response does not appear to be consistent with canonical mt-UPR.

Supplementary Methods: Normalization of Relative Expression in different RNA sequencing approaches

The problem of RNASeq data normalization has been extensively discussed (see, e.g., Ref. [7] and references therein). One of the key confounding issues underlying this problem is that NGS cDNA libraries are prepared and amplified by PCR, the efficiency and quality of which vary with the nucleotide sequence. As a result, the number of reads per cDNA fragment – which is the starting point for all analysis – may depend not only on the abundance of the corresponding mRNA but also on its sequence. This and other RNASeq technicalities discussed below are expected to have particularly strong effects on transcripts from low expression genes. Some of the data normalization procedures (e.g., LogNormalize and SCTransform for scRNASeq or DESeq2 for bulk RNASeq) attempt to address the resulting uncertainties in quantifying transcription of low expression genes through different mathematical means. However, these models have been developed based on RNASeq data from cell populations other than OBs. Therefore, we examined whether they are applicable to our study or other approaches to OBs were needed.

scRNASeq

In 10X Genomics scRNASeq and srRNASeq assays used for the present study, the PCR problem is partially resolved by utilizing 3' sequencing with UMIs (see manufacturer's protocols). Specifically, each cDNA is reverse transcribed from the 3' end of mRNA by capturing the mRNA vis hybridization with oligonucleotides that contain poly(dT) for the hybridization and unique molecular identifier (UMI) barcodes. This allows each mRNA molecule to be counted only once based on the UMI regardless of the number of copies produced by the PCR, reducing PCR artifacts. This partially solves the problem, at least for genes that produce enough mRNA for reliable reverse transcription and PCR amplification.

Nonetheless, 10X Genomics and all other UMI-based scRNASeq assays have another crucial limitation affecting data analysis. It is related to a finite number of the UMI-tagged oligonucleotides available for reverse transcription of mRNA from each cell, N_{UMI}. In the 10X Genomics assay, N_{UMI} is the number of oligonucleotides on each gel bead that is captured within an emulsion droplet with its cell. Because of the technicalities of the bead manufacturing process, N_{UMI} is inherently not the same for different beads (and therefore cells) and may be highly variable. It is one of the factors contributing to the large variation in the sequencing depth per cell.

Ideally, the goal of scRNASeq would be to accurately measure the actual number of transcripts for each gene *i* ($mRNA_i$) in each cell, but the current technology does not allow it. Indeed, when the total number of transcripts in a cell, $mRNA_{cell}$ becomes comparable to or exceeds N_{UMI}, the relationship between the number of reverse transcribed UMIs per gene *i* (UMI_i) and the number of reverse transcribed UMIs per cell ($UMI_{cell} = \Sigma_i UMI_i$) become nonlinear and dependent on N_{UMI}. Not only N_{UMI} varies from bead to bead, but $mRNA_{cell}$ varies from cell to cell. The resulting cell-to-cell variation in UMI_{cell} (further confounded by the sequencing depth) would be difficult to account for even if mRNA hybridization with the oligonucleotides followed an equilibrium binding isotherm. (The equilibrium binding is not likely either given high activation energy and therefore practically infinite time needed for equilibrating mRNA hybridization.) In other words, the general relationship between the measured relative count

$$RC_i = \frac{UMI_i}{UMI_{cell}} \tag{1}$$

and the actual fraction of the transcript in the cell ($mRNA_i / mRNA_{cell}$) is: (a) not linear, (b) not trivial, and (c) not known. Even if we were to assume that the subsequent PCR and sequencing were ideal, there

would be no statistically rigorous way to account for this effect without establishing the efficiency of hybridization of different mRNAs with the oligonucleotides, the statistics of N_{UMI}, and the statistics of $mRNA_{cell}$. Because of their large number, highly expressed genes get to "choose" the oligonucleotides once the cell is lysed. For these genes, the approximation $RC_i \approx (mRNA_i / mRNA_{cell})$ may be reasonable. For low and potentially even moderate expression genes, it may not be the case. To avoid overinterpreting and/or misinterpreting scRNASeq data, it is crucial to at least acknowledge this limitation.

With this in mind, %UMI used in the present study,

$$\%UMI_i = 100\% \cdot RC_i \tag{2}$$

provides the simplest possible approach to data normalization that accounts for all sources of variations in the sequencing depth (including N_{UMI}) without attempting to parametrize the unknown relationship between RC_i and $(mRNA_i / mRNA_{cell})$. A scaled relative count,

$$sRC_i = sf \cdot RC_i \tag{3}$$

is essentially the same approach implemented in Seurat, except it utilizes a scaling factor sf that can be set by the user (Seurat's default is $sf = 10{,}000$). For instance, $\%UMI_i$ can be used for estimating differential expression of the gene i between cells that have similar overall mRNA transcription ($mRNA_{cell}$), e.g., G610C OBs vs. WT OBs with the same level of *Col1a1* mRNA. In this case, the unknown relationship between the measured RC_i and the fraction of the transcript in the cell would be the same for the two types of cells and would not alter the conclusions.

Unfortunately, the same cannot be guaranteed for other normalization procedures that parametrize the data and incorporate additional model assumptions and/or nonlinear transformations, e.g., SCTransform and LogNormalize procedures in Seurat. Regardless of potential benefits of these procedure for some applications, we do not find them to be justified for differential gene expression analysis in OBs and potentially other highly secretory cells.

A key feature of mature OBs (mOBs) is that collagen I mRNA (*Col1a1* + *Col1a2*) overwhelms all other transcripts, accounting for up to 40% of all mRNA molecules detected by scRNASeq in the cell (Supp. Figure 7A). To keep up with the massive collagen synthesis, the cell must selectively ramp up transcription of multiple other genes (e.g., ribosomal proteins), resulting in at least ~ 2-fold increase in the total mRNA/cell during OB maturation (Supp. Figure 7B-D). In the end, mOBs may produce ~ 5 times more mRNA/cell than non-secretory cells (e.g., macrophages, Supp. Figure 7B). As noted above, this feature of mRNA transcription in mOBs does not affects the analysis based on nonparametric normalization with $\%UMI_i$ (or sRC_i), yet it is incompatible with models based on nonlinear transformations or existing parametrized models of data dispersion.

For instance, consider the SCTransform data parametrization model implemented in Seurat, which is rapidly gaining popularity.[8] In SCTransform, the data are normalized by extracting the normalization parameters from negative binomial regression of UMI_i vs. UMI_{cell} (followed by subsequent regularization to avoid data overfitting). Regardless of the general merits of *ad hoc* assumptions built into this approach, such parametrization may severely distort differential expression results for mOBs because it is based on regression across all cells rather than just the cells with the same mRNA transcription patterns (e.g., $mRNA_{cell}$). As discussed above, just a change in the $mRNA_{cell}$ should already be expected to alter the relationship between UMI_i and the actual transcript fraction in the cell (as well as between UMI_i and UMI_{cell}). In our opinion, the ~ 5-fold or larger difference in $mRNA_{cell}$ between OBs and other cells and at least 2-fold difference in $mRNA_{cell}$ within the OB subpopulation (Supp. Figure 7B) preclude utilization of SCTransform for analyzing differential expression of low and moderately expressed genes in our case.

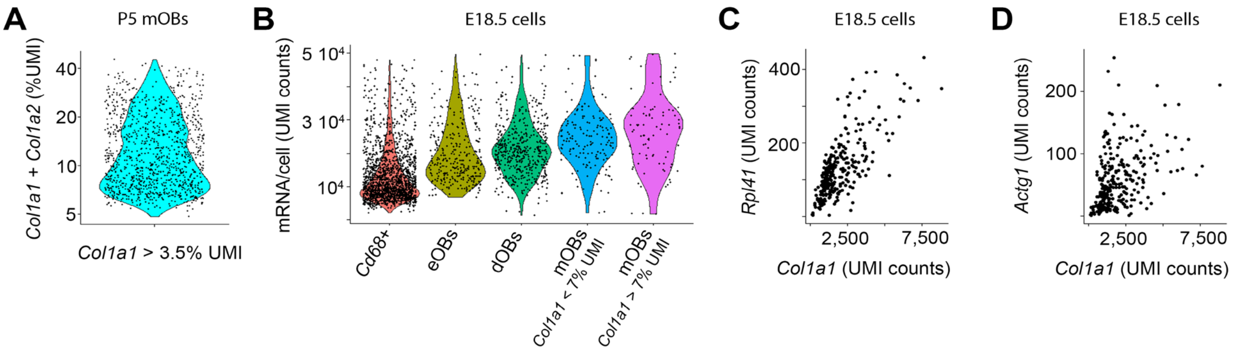

Supplementary Figure 7. Transcription of mRNA in OBs. **A.** *Col1a1*+*Col1a2* fraction of total mRNA molecules in mOBs (defined as OBs with *Col1a1* > 3.5% UMI, see Fig. 2A). **B.** Dependence of the total mRNA/cell on the level of collagen synthesis (eOBs = *Col1a1* ≤ 0.75% UMI, dOBs = 0.75% < *Col1a1* ≤ 3.5 % UMI, see Fig. 2A). *Cd68*+ cells are monocytes (mostly macrophages) with Cd68 > 3 (UMI count). **C.** Correlation between the amount *Rpl41* mRNA (encoding ribosomal protein L41 essential for collagen translation) and *Col1a1* mRNA in mOBs. **D.** Correlation between *Actg1* mRNA (encoding actin G1) and *Col1a1* mRNA. A strong correlation between *Col1a1* and gene transcripts involved in collagen synthesis combined with the lack of such correlation for other transcripts indicates that OBs selectively ramp up transcription of the former genes to support collagen production.

Note that E18.5 cells were used for panels B-D because of inherent limitations on N_{UMI} in the 10X Genomics scRNASeq assay we utilized. In this assay, $mRNA_{cell}$ was not only larger than N_{UMI} for mOBs but also close to N_{UMI} even in those P5 cells that produced little or no collagen (~ 30,000 transcripts per average macrophage). As a result, we could detect the change in $mRNA_{cell}$ only in E18.5 cells (<10,000 transcripts per average macrophage and <20,000 transcripts per average eOB).

Another approach implemented in various scRNASeq data analysis packages is logarithmic normalization. For instance, the default Seurat procedure is LogNormalize, in which the log-normalized gene expression (LNE_i) for gene i is calculated from the raw UMI_i count for the gene as

$$LNE_i = \ln(1 + sf \cdot RC_i), \qquad (4)$$

e.g., to avoid ln(0). For differential gene expression analysis in Seurat v4 (FindMarkers function), this normalization involves another adjustable parameter pc (pseudocount) that can be set by the user (Seurat's default is $pc = 1$), which effectively replaces 1 in a logarithm similar to Eq. (4) when calculating mean fold-change in gene expression. The choice of sf and pc allows the user to suppress variations in LNE_i for low expression genes, but fold-change in gene expression then becomes dependent on both sf and pc. Whatever the benefits of LogNormalize might be, RC_i in our data set varies from $RC_i \sim 2\cdot10^{-5}$ at $UMI_i = 1$ and $UMI_{cell} \sim 50{,}000$ to $RC_i \sim 0.1$ at $UMI_i \sim 500$ and $UMI_{cell} \sim 5{,}000$, causing strongly nonlinear dependence of fold-change in expression of most genes on sf and pc. Not only the results become highly dependent on the choice of sf and pc, but the effects of this choice become very different for genes with different expression level. The dependence on sf and pc can be eliminated only by setting $sf >> \max(pc \cdot UMI_{cell})$, but that is an arbitrary choice as well. Without a validated justification for the choice of sf and pc that is based on objective criteria grounded in experimental facts rather than on the desired appearance of the results, such an approach may not be advisable. Unfortunately, we are not aware of such a justification.

Therefore, we normalized and reported all data as %UMI to avoid biasing the outcome of our analysis.

Bulk RNASeq

Like SCTransform, the DESeq2 approach claims to address the data normalization problem in bulk RNASeq through advanced mathematical modeling and regression procedures based on *ad hoc* assumptions (e.g., the negative binomial model).[9] In our opinion, utilization of this approach for special cell populations like OBs may not be advisable for essentially the same reasons as SCTransform. When combined with PCR-related effects and inherent lack of a linear relationship between expression of some genes and $mRNA_{cell}$, large systematic rather than random variations in total $mRNA_{cell}$ (Supp. Figure 7B) may introduce even bigger problems for data parametrization. The assumptions built into DESeq2 are inconsistent with our data set and may thereby lead to unreliable analysis outcome.

In other common data normalization procedures used for traditional bulk RNASeq, e.g., FPKM and TPM, the PCR problem is ignored for the lack of a better solution.[10] FPKM and TPM are not based on data parametrization, but these approaches may also be problematic in our case because of PCR bias. Indeed, as shown in Supp. Figure 7A, mOBs – our main cells of interest – have disproportionately high content of *Col1a1* and *Col1a2* mRNA. The corresponding cDNA is rich in GC base pairs and repetitive sequences, presenting a challenge for PCR. Under these conditions, reduced transcription of *Col1a1* and *Col1a2* by Het vs WT cells (Figure 2) may introduce a systematic bias into the analysis.

To verify whether this is indeed the case, we compared expression of several housekeeping genes in Het and WT cells using just the minimally necessary normalization for the sequencing depth. First, we utilized our scRNASeq dataset for P5 OBs to choose and validate housekeeping genes that are truly independent of the OB differentiation stage. We chose this dataset because the cultured primary cells were extracted from the same P5 parietal bones as used for the scRNASeq experiments.

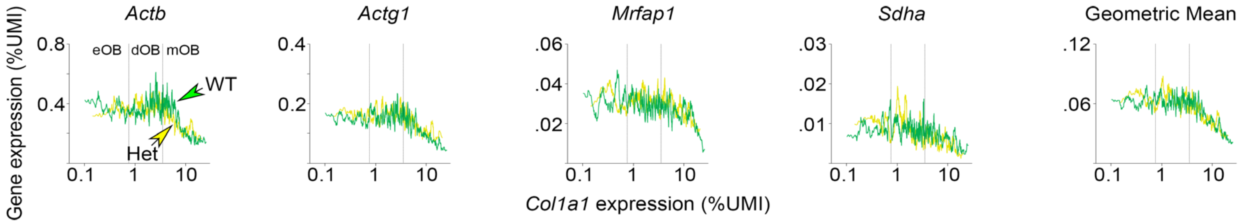

Supplementary Figure 8. Housekeeping genes. The plots show dependence of housekeeping gene expression in Het (yellow) and WT (green) cells on *Col1a1* expression determined by scRNASeq of P5 parietal bone cells (20-cell running average calculated as described in Fig. 3B). Geometric Mean is the geometric mean for the 4 genes.

After examining ~ 50 housekeeping gene candidates previously studied by others,[11] we found that *Actg1*, *Actb*, *Mrfap1*, and *Sdha* met all the criteria (Supp. Figure 8). (a) Their expression was the same in Het and WT OBs at the same level of *Col1a1* expression. (b) Their %UMI remained constant at low *Col1a1* expression and decreased at high *Col1a1* expression in mOBs, which was expected for genes unrelated to collagen synthesis once the total mRNA/cell began to increase due to increasing *Col1a1* transcription. (c) The relative dispersion of %UMI for these genes was within the average range for OBs.

We then confirmed the Het vs. WT bias in bulk RNASeq by observing that the geometric mean of the depth-normalized expression for *Actg1*, *Actb*, *Mrfap1*, and *Sdha* in bulk RNASeq was noticeably higher in Het vs WT cells (Supp. Figure 9, last panel). Like the genes themselves, the geometric mean %UMI for these genes exhibited no difference in %UMI between Het and WT at the same level of *Col1a1*

expression (Supp. Figure 8). We also observed the same bias for each of the 4 genes individually (Supp. Figure 9). As noted above, this bias could be caused by higher PCR quality and efficiency because of the lower average *Col1a1* mRNA content in Het cells (Supp. Table 1). The bias could also be a simple arithmetic consequence of lower average $mRNA_{cell}$ in Het vs. WT and therefore higher normalized counts for all genes in Het OBs.

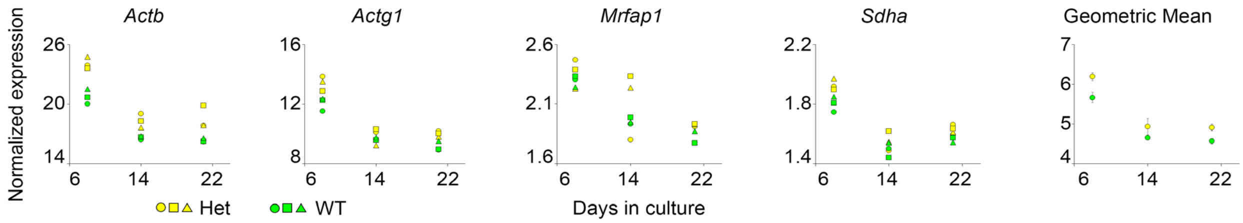

Supplementary Figure 9. Bias in housekeeping gene expression after sequencing depth normalization of bulk RNASeq. The plots show dependence of housekeeping gene expression in primary cells isolated from P5 parietal bones on days in culture determined by bulk RNASeq (expression was normalized to sequence depth and scaled). Circles, squares, and triangles show 3 Het (yellow) and 3 WT (green) replicates. Error bars in the geometric mean plot show standard deviation for the 3 replicates. The observed bias is caused by lower average *Col1a1* expression in Het vs. WT cells. Unlike scRNASeq in Supp. Figure 8, this bias cannot be removed by data stratification based on *Col1a1* expression in individual cells since such information is not available in bulk RNASeq.

To resolve this bias problem, we utilized a well-tested empirical solution known since the early days of qPCR, which is normalizing the data with the geometric mean of housekeeping gene expression. Specifically, we divided the sequencing-depth-normalized counts for the genes of interest by the geometric mean of the housekeeping gene counts, yielding expression values relative to the housekeeping genes. By utilizing the same, validated *Actg1*, *Actb*, *Mrfap1*, and *Sdha* genes, we could then compare the expression of various genes in Het and WT cells similar to the $\Delta\Delta C_T$ approach in qPCR. Additional normalization for the gene length (akin to FPKM) could be utilized as well, but it was not necessary in the context of our study since we were interested only in comparing relative expression of the same gene in Het and WT rather than in comparing relative expression of different genes. Given that this normalization was not needed and that it would be based on a questionable assumption of similar PCR efficiency across the gene length and between different genes, we decided to avoid it.

References

[1] Y. Hao, S. Hao, E. Andersen-Nissen, W. M. Mauck, 3rd, S. Zheng, A. Butler, M. J. Lee, A. J. Wilk, C. Darby, M. Zager, P. Hoffman, M. Stoeckius, E. Papalexi, E. P. Mimitou, J. Jain, A. Srivastava, T. Stuart, L. M. Fleming, B. Yeung, A. J. Rogers, J. M. McElrath, C. A. Blish, R. Gottardo, P. Smibert, R. Satija, *Cell* **2021**, *184* (13), 3573, https://doi.org/10.1016/j.cell.2021.04.048.

[2] L. S. Mirigian, E. Makareeva, E. L. Mertz, S. Omari, A. M. Roberts-Pilgrim, A. K. Oestreich, C. L. Phillips, S. Leikin, *J Bone Miner Res* **2016**, *31* (8), 1608, https://doi.org/10.1002/jbmr.2824.

[3] C. Hetz, K. Zhang, R. J. Kaufman, *Nat Rev Mol Cell Biol* **2020**, *21* (8), 421, https://doi.org/10.1038/s41580-020-0250-z.

[4] S. Takayanagi, R. Fukuda, Y. Takeuchi, S. Tsukada, K. Yoshida, *Cell Stress Chaperones* **2013**, *18* (1), 11, https://doi.org/10.1007/s12192-012-0351-5.

[5] L. Sampieri, P. Di Giusto, C. Alvarez, *Front Cell Dev Biol* **2019**, *7*, 123, https://doi.org/10.3389/fcell.2019.00123.

[6] S. Ito, K. Nagata, *J Biol Chem* **2019**, *294* (6), 2133, https://doi.org/10.1074/jbc.TM118.002812.

[7] S. Choudhary, R. Satija, *Genome Biol* **2022**, *23* (1), 27, https://doi.org/10.1186/s13059-021-02584-9.
[8] C. Hafemeister, R. Satija, *Genome Biol* **2019**, *20* (1), 296, https://doi.org/10.1186/s13059-019-1874-1.
[9] M. I. Love, W. Huber, S. Anders, *Genome Biol* **2014**, *15* (12), 550, https://doi.org/10.1186/s13059-014-0550-8.
[10] Y. Zhao, M. C. Li, M. M. Konate, L. Chen, B. Das, C. Karlovich, P. M. Williams, Y. A. Evrard, J. H. Doroshow, L. M. McShane, *J Transl Med* **2021**, *19* (1), 269, https://doi.org/10.1186/s12967-021-02936-w.
[11] M. Caracausi, A. Piovesan, F. Antonaros, P. Strippoli, L. Vitale, M. C. Pelleri, *Mol Med Rep* **2017**, *16* (3), 2397, https://doi.org/10.3892/mmr.2017.6944.
